# Molecular mechanism of the MORC4 ATPase activation

**DOI:** 10.1038/s41467-020-19278-8

**Published:** 2020-10-29

**Authors:** Adam H. Tencer, Khan L. Cox, Gregory M. Wright, Yi Zhang, Christopher J. Petell, Brianna J. Klein, Brian D. Strahl, Joshua C. Black, Michael G. Poirier, Tatiana G. Kutateladze

**Affiliations:** 1 Department of Pharmacology, University of Colorado School of Medicine, Aurora, CO 80045 USA; 2 Department of Physics, Ohio State University, Columbus, OH 43210 USA; 3 Department of Biochemistry & Biophysics, the University of North Carolina School of Medicine, and UNC Lineberger Comprehensive Cancer Center, Chapel Hill, NC 27599 USA

**Keywords:** Enzyme mechanisms, Structural biology, Nucleosomes, X-ray crystallography

## Abstract

Human Microrchidia 4 (MORC4) is associated with acute and chronic pancreatitis, inflammatory disorders and cancer but it remains largely uncharacterized. Here, we describe the structure–function relationship of MORC4 and define the molecular mechanism for MORC4 activation. Enzymatic and binding assays reveal that MORC4 has ATPase activity, which is dependent on DNA-binding functions of both the ATPase domain and CW domain of MORC4. The crystal structure of the ATPaseCW cassette of MORC4 and mutagenesis studies show that the DNA-binding site and the histone/ATPase binding site of CW are located on the opposite sides of the domain. The ATPase and CW domains cooperate in binding of MORC4 to the nucleosome core particle (NCP), enhancing the DNA wrapping around the histone core and impeding binding of DNA-associated proteins, such as transcription factors, to the NCP. In cells, MORC4 mediates formation of nuclear bodies in the nucleus and has a role in the progression of S-phase of the cell cycle, and both these functions require CW and catalytic activity of MORC4. Our findings highlight the mechanism for MORC4 activation, which is distinctly different from the mechanisms of action observed in other MORC family members.

## Abstract (web summary)

Human Microrchidia 4 (MORC4) ATPase has been implicated in acute and chronic pancreatitis, inflammatory disorders and cancer. Here the authors describe the structure–function relationship of MORC4 and define the molecular mechanism for MORC4 activation.

## Introduction

Microrchidia 4 (MORC4) is a poorly characterized member of the new family of CW-type zinc finger nuclear proteins. MORC4 has been associated with acute and chronic pancreatitis and inflammatory bowel disorders, including Crohn’s disease and ulcerative colitis, and more recently was found to be overly expressed in breast cancer cells and in diffuse large B cell lymphoma^1–8^. Analysis of breast cancer tissues shows that MORC4 is negatively regulated by microRNAs, including miR-193b-3p, and elevated MORC4 levels are linked to poor survival^9^. Despite growing evidence suggests an essential role of MORC4 in normal physiological and pathological processes, how this protein functions at molecular and cellular level is not well understood.

**Fig. 1:**
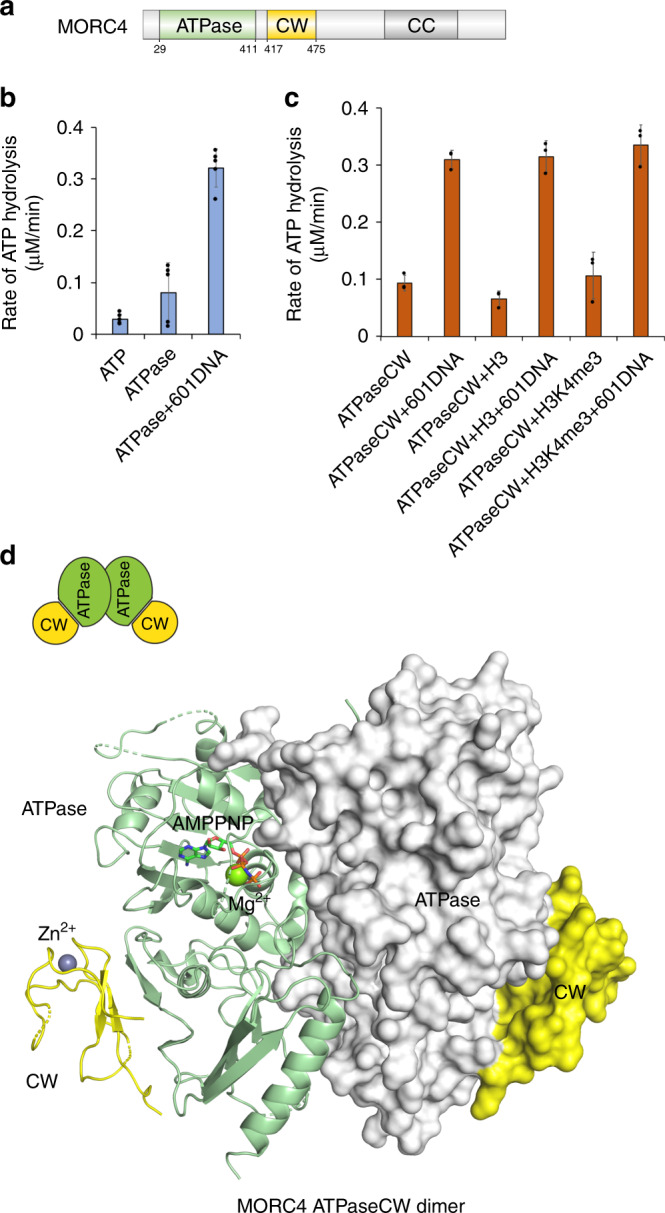
MORC4 is an ATPase. **a** MORC4 domain architecture. The ATPase and CW domains are colored green and yellow, respectively. The ATPaseCW cassette construct contains aa 29–486 of MORC4 (Q8TE76). **b**, **c** Rates of ATP hydrolysis by the ATPase domain of MORC4 (**b**) and the ATPase-CW cassette of MORC4 (**c**). Data are represented as mean values +/− S.D. of at least three independent experiments (*n* ≥ 3). Source data are provided in a Source Data file. **d** The crystal structure of the dimeric MORC4 ATPase-CW/AMPPNP complex. In protomer A, the ATPase domain and the CW domain are shown in a ribbon diagram and colored green and yellow, respectively. In protomer B, the surface representation of the ATPase domain (white) and the CW domain (yellow) is shown. The magnesium (green) and zinc (gray) atoms are shown as spheres. The AMPPNP molecule is in stick representation. Dashed lines represent residues and loops that are not defined by electron density.

Members of the MORC family are characterized by their gyrase, Hsp90, histidine kinase, and MutL (GHKL)-type ATPase domain, which is also present in several chromatin-modifying enzymes^10,11^. Although the ability of MORC4 to hydrolyze ATP has not been investigated, the homologous MORC proteins, such as MORC2 and MORC3 are enzymatically active. MORC3 has been shown to exist in an autoinhibited state and is activated through binding to methylated histone mark H3K4me3, associated with transcriptionally active chromatin^12–16^. Conversely, MORC2 does not recognize H3K4me3 and is necessary for the human silencing hub (HUSH)-dependent silencing of transgenes integrated at chromatin loci with the methylated histone mark H3K9me3^17,18^. The ATPase domain of MORCs is followed by a CW-type zinc finger and one or more coiled-coil regions (Fig. 1a). Recognition of H3K4me3 by MORC3 is mediated by the CW domain, and this interaction regulates the catalytic activity of MORC3 and is required for the MORC3 recruitment to chromatin and formation of liquid–liquid phase separation (LLPS) droplets in the nucleus^13,15,19,20^. While we have begun understanding the markedly different mechanisms of action and some biological roles of the individual MORC2 and MORC3 proteins, virtually nothing is known about the function of MORC4.

Here, we describe the unique molecular mechanism for MORC4 activation. We show that MORC4 is an ATPase with an intrinsic DNA-dependent but histone-independent enzymatic activity. The ATPase and CW domains of MORC4 cooperate in binding of MORC4 to the NCP, impeding the association of transcription factors with the NCP. Our cell data reveal two biological functions of MORC4: it regulates the formation of nuclear bodies (NBs) in the nucleus and plays a role in the cell cycle S-phase progression with the ATPase activity and chromatin binding being necessary for both functions.

## Results

### MORC4 is an ATPase

To determine whether MORC4 is catalytically active, we produced the ATPase domain of human MORC4 and examined its ability to hydrolyze ATP in enzymatic assays (Fig. 1a–c). Following incubation of the ATPase domain with ATP, 2-amino-6-mercapto-7-methylpurine ribonucleoside and nucleoside phosphorylase, the release of inorganic phosphate generated by the hydrolysis of ATP to ADP was monitored by measuring an increase in absorbance at 360 nm. We found that the MORC4 ATPase domain itself possesses little ATP hydrolyzing activity, however addition of 147 base pair (bp) 601 Widom DNA at a 1:1 molar ratio led to a ~4-fold increase in the rate of ATP hydrolysis, indicating that the catalytic activity of the ATPase domain is DNA-dependent (Fig. 1b). A similar increase in the ATP hydrolysis rate was observed upon adding 601 DNA to the ATPase domain linked to the CW domain of MORC4 in the ATPaseCW cassette (Fig. 1a, c). Neither methylated histone peptide H3K4me3 nor unmodified H3 peptide (both aa 1–12 of H3) stimulated a further increase in the rate of ATP hydrolysis, implying that the enzymatic activity is largely histone independent (we discuss binding of CW to H3 below). The nucleosome core particle (NCP) also stimulated the catalytic activity of the ATPaseCW cassette (Supplementary Fig. 1). Together, these data demonstrate that the ATPase activity is a function of MORC4, which requires the presence of DNA.

### Structure of the MORC4 ATPaseCW cassette

To gain mechanistic insight into the MORC4 activation process, we crystallized ATPaseCW (aa 29–486 of human MORC4) in complex with the non-hydrolyzable ATP analog, adenylyl-imidodiphosphate (AMPPNP), collected and analyzed X-ray crystallographic data, and refined the structure to 2.9 Å resolution (Fig. 1d and Supplementary Table 1). The structure reveals a symmetric parallel homodimer with each ATPaseCW protomer coordinating one molecule of AMPPNP and one Mg^2+^ ion. The ATPase module folds into the GHKL-type domain, consisting of a mixture of α-helices and β-strands, and is involved in the formation of a large dimer interface, which was also observed in the respective structures of MORC3 and MORC2^14,15,17^. Two zinc-binding CW domains tightly pack against the ATPase domains and are positioned on the sides of the ATPase domains opposite to the dimer interface and are far apart (colored yellow in Fig. 1d).

Each CW domain interacts mainly with the loops between α8 and β10 of the ATPase domain (Supplementary Fig. 2). The residues P279, K289, and K297 of the ATPase domain form a number of polar and electrostatic contacts with the Q424, W426, Q428, D430, W435, and A449 residues of the CW domain. In addition, the hydrophobic side chain of M294 is caged between the aromatic rings of W426 and W435 of CW, and the side chain of R69 of the ATPase domain is restrained by hydrogen bonds with the backbone carbonyl groups of D430 and E431 and the carboxyl group of E431 of CW.

Strikingly, while the MORC4 ATPaseCW cassette structure superimposes well with the structure of ATPaseCW in the homologous protein MORC3^15^ (RMSD of 1.5 Å) and shares high sequence similarity with MORC3 (Supplementary Fig. 3), the mechanisms of action of the two proteins differ. The ATPaseCW cassette of MORC4 is capable of binding to DNA and therefore is active, whereas the ATPaseCW cassette of MORC3 represents an autoinhibited state, which does not inherently bind DNA. Binding of the CW domain of MORC3 to histone H3K4me3, which leads to the disruption of the CW:ATPase complex^14^ (the same surface of CW is involved in binding to the ATPase domain and H3K4me3^13–15^), is required for the release of autoinhibition, thus allowing interaction with DNA and resulting in MORC3 activation^13,15^. Why is the catalytic activity of MORC4 independent of histone binding in contrast to the MORC3 catalytic activity and what does then stimulate MORC4 activation? To explain this, we considered three possibilities, i.e., MORC4 does not bind histone tails, DNA binding occludes the histone-binding site, or DNA binding promotes the catalytic activity of MORC4 independently of histone binding.

### The CW domain of MORC4 selects for histone H3K4me3

**Fig. 2:**
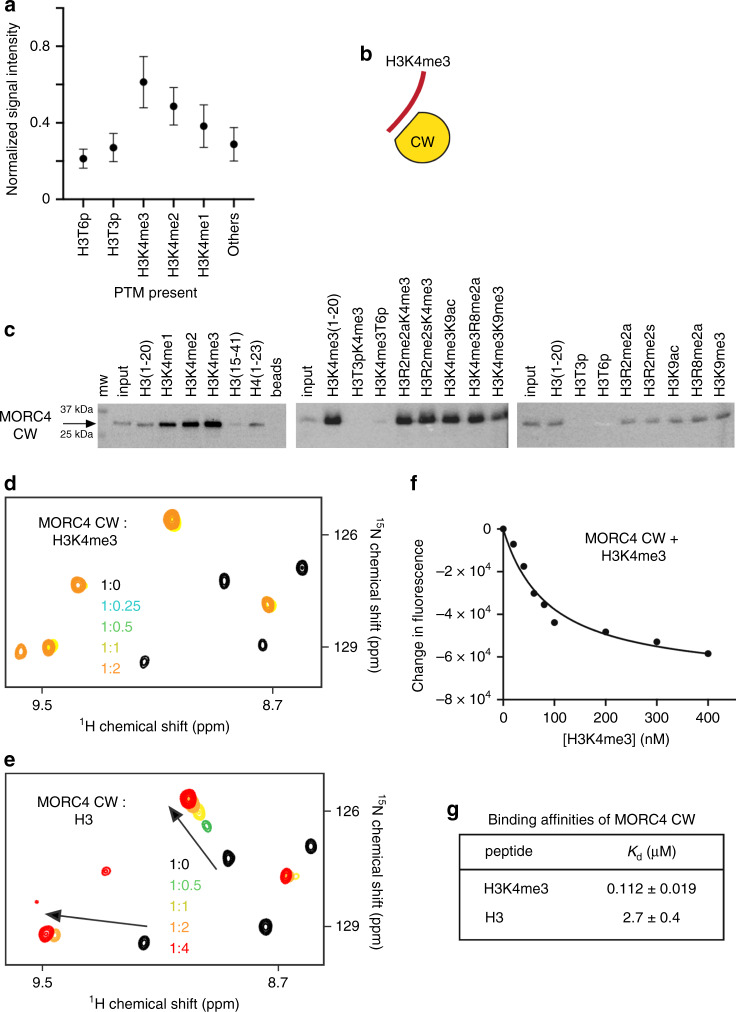
The CW domain of MORC4 recognizes H3K4me3. **a** Normalized average microarray signal intensities detected upon binding of the GST-tagged MORC4 CW domain to the peptides carrying indicated PTMs. Data are represented as mean values +/− S.D. from *n* ≥ 3 independent arrays. See also Supplementary Data 1. **b** Cartoon representation of CW in complex with histone H3K4me3 tail. **c** Peptide pull-down assays of the GST-tagged MORC4 CW domain using the indicated histone H3 peptides. Images are representative of three independent experiments with similar results (*n* = 3). Source data are provided in a Source Data file. **d**, **e** Superimposed ^1^H,^15^N HSQC spectra of the ^15^N-labeled WT MORC4 CW domain collected upon titration with the histone H3K4me3 (**d**) and unmodified H3 (**e**) peptides. Spectra are color coded according to the protein-to-peptide molar ratio. **f** Representative binding curve used to determine *K*_d_ values by tryptophan fluorescence. **g** Binding affinities of MORC4 CW for the indicated histone H3 peptides measured by tryptophan fluorescence. Data are represented as mean values +/− S.D. from three independent experiments (*n* = 3). Source data are provided in a Source Data file.

A set of zinc fingers, including the CW domain of MORC3^13^, has been shown to recognize histone sequences, therefore we tested whether the MORC4 CW domain either isolated (in this section) or linked to the ATPase domain in the ATPaseCW cassette (below) is capable of binding to histone tails using high-throughput histone peptide microarrays, pull-down assays and NMR (Fig. 2). In microarrays, GST-tagged CW was incubated with a library of over ~250 synthetic histone peptides containing single posttranslational modifications (PTMs) or combinations of PTMs, including acetylated and methylated lysine residues, phosphorylated serine and threonine residues and methylated arginine residues known to be present in the core and variant histone proteins (Supplementary Data 1). We found that the MORC4 CW domain binds to the N-terminal histone H3 peptides, selecting for methylated H3K4 sequences and does not recognize histone H4, H2A, and H2B (Fig. 2a, b). These results were corroborated by pull-down assays, in which GST-tagged CW domain of MORC4 was incubated with biotinylated unmodified and modified histone H3 peptides. As shown in Fig. 2c, CW associated with methylated H3K4 peptides, preferring the higher (tri)methylation state, H3K4me3. While methylation of the neighboring sites H3R2 and H3R8 appeared to not affect binding of CW to H3K4me3, phosphorylation of H3T3 or H3T6 disrupted interaction with the doubly modified H3T3phK4me3 and H3K4me3T6ph peptides.

To further compare binding of the MORC4 CW domain to methylated and unmodified H3K4, we produced ^15^N-labeled CW and examined it in ^1^H,^15^N heteronuclear single quantum coherence (HSQC) experiments (Fig. 2d, e). Titration of the H3K4me3 peptide (aa 1–12 of H3) resulted in substantial chemical shift perturbations (CSPs) in the MORC4 CW domain, indicating direct interaction. Throughout the gradual addition of the peptide, a number of crosspeaks corresponding to the free state of the protein disappeared, and simultaneously, another set of resonances, corresponding to the bound state, appeared. This pattern of CSPs indicates a tight binding in the slow exchange regime on the NMR time scale. However, interaction of the MORC4 CW domain with the unmodified H3 peptide was weaker judging by CSPs in the intermediate exchange regime. In support, dissociation constants (*K*_d_s) for the complexes of the MORC4 CW domain with the H3K4me3 and H3K4 peptides were found to be ~0.1 and 2.7 μM, respectively, as measured by tryptophan fluorescence (Fig. 2f, g and Supplementary Fig. 4). These results demonstrate that the MORC4 CW domain is not only a selective reader of H3K4me3 but also one of the strongest effectors of H3K4me3, which exhibits ~7–10 fold higher binding affinity to this PTM than other readers^21,22^.

**Fig. 3:**
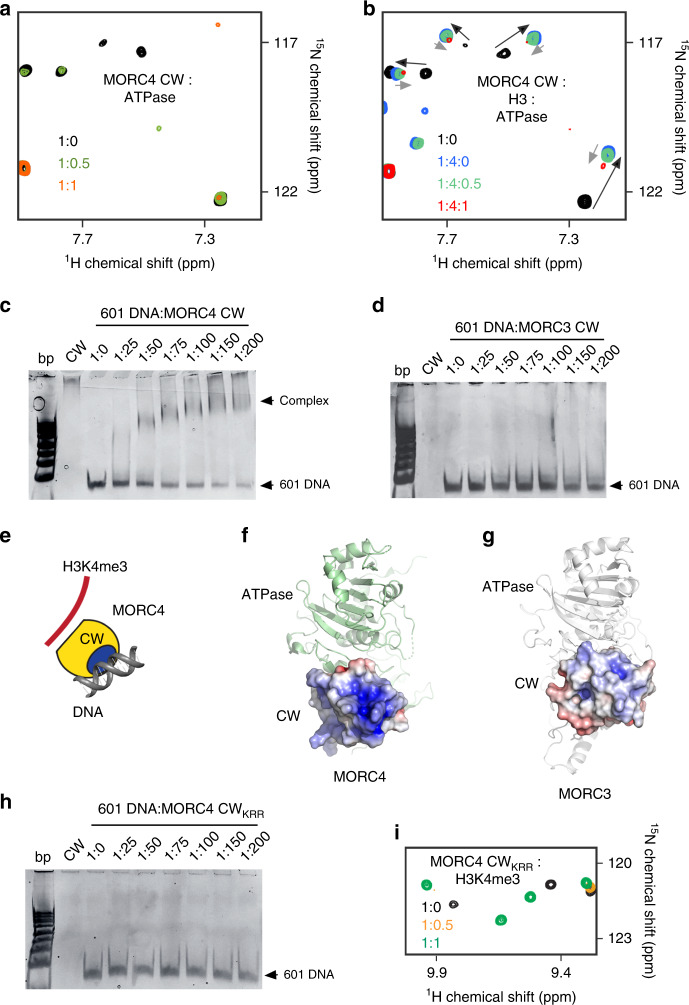
MORC4 CW binds to DNA. **a** Superimposed ^1^H,^15^N HSQC spectra of the ^15^N-labeled MORC4 CW domain collected upon titration with the unlabeled ATPase domain of MORC4. **b** Superimposed ^1^H,^15^N HSQC spectra of the ^15^N-labeled MORC4 CW domain (in complex with H3 peptide) collected upon titration with the unlabeled ATPase domain. Spectra in (**a**, **b**) are color coded according to the protein-to-ligands molar ratio. (**c**, **d**) EMSA with 601 DNA in the presence of increasing amounts of MORC4 CW (**c**) and MORC3 CW (**d**). **e** Cartoon representation of MORC4 CW in complex with histone H3K4me3 tail and DNA. **f**, **g** Electrostatic surface potential of the CW domain of MORC4 (**f**) and MORC3 (**g**) (within the ATPase-CW cassette) was generated using APBS in Pymol with a range of –5/5 kT/e and colored blue and red for positive and negative charges, respectively. The ATPase domain is shown as ribbon and colored green and white in MORC4 and MORC3, respectively. The missing loops (residues K441 and R463) in the model of MORC4 CW were completed for this figure. **h** EMSA with 601 DNA in the presence of increasing amounts of mutant MORC4 CW_KRR_. **i** Superimposed ^1^H,^15^N HSQC spectra of the ^15^N-labeled MORC4 CW_KRR_ domain collected upon titration with the histone H3K4me3 peptide. Spectra are color coded according to the protein-to-peptide molar ratio.

Furthermore, consecutive NMR titration experiments showed that the two ligands of the MORC4 CW domain—the ATPase domain and histone H3—compete for binding to CW. Gradual addition of the isolated unlabeled ATPase domain to the ^15^N-labeled MORC4 CW domain led to a decrease in intensities of amide resonances, which suggested formation of the large ATPase:CW complex (Fig. 3a). Titration of the ATPase domain into the CW:H3 complex caused CSPs that were indicative of the disruption of the CW:H3 complex and formation of the complex between CW and the ATPase domain (Fig. 3b). We concluded that MORC4 CW is capable of binding to either H3 or the ATPase domain and that, similarly to MORC3^13–15^, the ATPase-binding site and the histone-binding site of CW overlap.

### The MORC4 CW domain binds DNA

Considering the ability of the MORC4 ATPaseCW cassette to be activated by DNA, we investigated whether the CW domain contributes to the DNA binding by testing it in electrophoretic mobility shift assay (EMSA) (Fig. 3c). 601 DNA was incubated with increasing concentrations of CW, and the reaction mixtures were resolved on a 5% native polyacrylamide gel. A gradual increase in the amount of added CW resulted in the shift and disappearance of the free 601 DNA band and appearance of a band smear corresponding to the complex of CW with 601 DNA, indicating the direct interaction (Fig. 3c, e). Unlike the CW domain of MORC4, the CW domain of homologous MORC3 was largely incapable of binding to 601 DNA, as no DNA band shift was observed in EMSA with MORC3 CW (Fig. 3d).

To identify the DNA-binding site of the MORC4 CW domain, we examined its electrostatic surface potential (ESP) and compared it to that of the MORC3 CW domain (Fig. 3f, g). Analysis of ESP of MORC4 CW revealed that particularly one side of CW, which is opposite to the H3K4me3- and ATPase-binding sites, is highly positively charged. Specifically, K460, R462 and R463 form a well-defined positively charged cluster on the protein surface, which suggested to us that these residues might be involved in binding to the negatively charged DNA. We generated the triple mutant of the MORC4 CW domain, K460A/R462A/R463A (CW_KRR_), and tested its interaction with 601 DNA by EMSA. We did not observe the 601 DNA band shift even at the DNA to CW_KRR_ molar ratio of 1 to 200 (Fig. 3h), whereas H3K4me3 binding activity of this mutant appeared to be unaffected based on the pattern of CSPs seeing in NMR titration experiment with the H3K4me3 peptide (Fig. 3i). Collectively, these data suggest that the K460, R462, and R463 residues of the CW domain of MORC4 are required for the strong interaction with DNA and that the DNA-binding site and the histone/ATPase-binding sites are located on the opposed sides of the MORC4 CW domain.

### DNA-binding activity of the MORC4 CW domain is essential

**Fig. 4:**
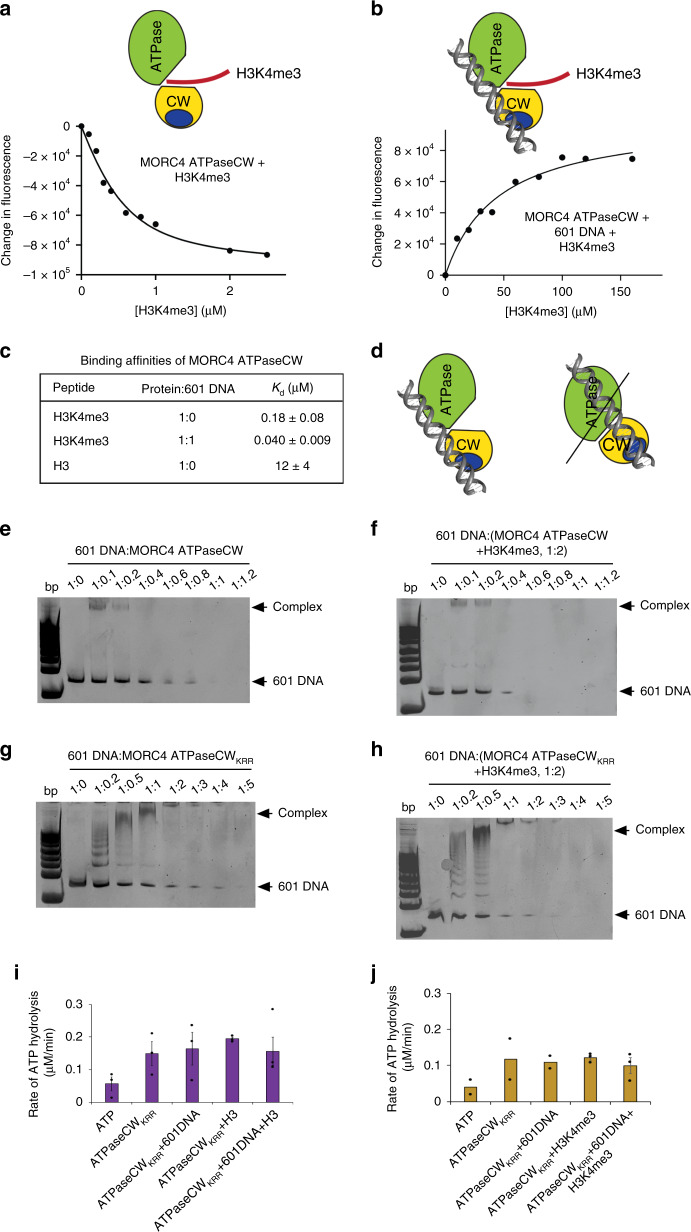
DNA binding by MORC4 CW is necessary for ATP hydrolysis. **a**, **b** Representative binding curves used to determine *K*_d_ values by fluorescence. Cartoon representations of MORC4 ATPaseCW binding to histone H3 tail in the absence (**a**) and presence (**b**) of DNA are shown above the curves. **c** Binding affinities of MORC4 ATPaseCW for the indicated histone H3 peptides (with or without 601 DNA) measured by tryptophan fluorescence. Data are represented as mean values +/− S.D. from three independent experiments (*n* = 3). Source data are provided in a Source Data file. **d** Models of the MORC4 ATPaseCW-DNA complex. **e**–**h** EMSA with 601 DNA in the presence of increasing amounts of WT (**e**, **f**) and mutant (**g**, **h**) MORC4 ATPaseCW in the absence (**e**, **g**) and presence (**f**, **h**) of histone H3K4me3 peptide. Experiments in (**e**) and (**g**) were repeated ten times and two times, respectively. **i**, **j** Rates of ATP hydrolysis by mutant MORC4 ATPaseCW_KRR_ in the presence of histone H3 (**i**) or histone H3K4me3 peptide (**j**). Data are represented as mean values +/− S.D. of three independent experiments. Error was not calculated for the first three data sets containing two data points in (**j**) (*n* = 2). Source data are provided in a Source Data file.

Can binding of the ATPaseCW cassette to DNA occlude the histone-binding site? We tested this possibility by measuring binding affinities of the ATPaseCW cassette for the H3 peptides in the absence and presence of 601 DNA by tryptophan fluorescence (Fig. 4a–c). We found that the ATPaseCW cassette binds to H3K4me3 only slightly (and not significantly) weaker as compared to binding of the isolated CW domain to the same H3K4me3 peptide (*K*_d_s of 180 nM and 112 nM, respectively) (Figs. 2f, g and 4a, c and Supplementary Fig. 4). However, interaction of the ATPaseCW:601 DNA complex with H3K4me3 peptide was ~4.5-fold stronger (*K*_d_ of 40 nM), implying that DNA binding enhances rather than impedes binding to H3K4me3 (Fig. 4d). These results led us to the current model for the MORC4 activation, in which DNA binding by the ATPaseCW cassette (both ATPase and CW domains are involved in this interaction) promotes the catalytic activity and binding of CW to H3K4me3 (Fig. 4d). This model was further corroborated by EMSA experiments (Fig. 4e–h). First, the presence of H3K4me3 peptide slightly increased binding of either wild-type ATPaseCW or mutated ATPaseCW_KRR_ to 601 DNA, and second, the DNA-binding function of the mutated ATPaseCW_KRR_ cassette was notably reduced, demonstrating the role of CW in the association with DNA. The importance of the DNA-binding activity of CW in activation of MORC4 was underscored by the fact that the mutant ATPaseCW_KRR_ cassette failed to be stimulated to hydrolyze ATP in enzymatic assays (Fig. 4i, j).

### Extensive DNA-binding site of the MORC4 ATPaseCW cassette

**Fig. 5:**
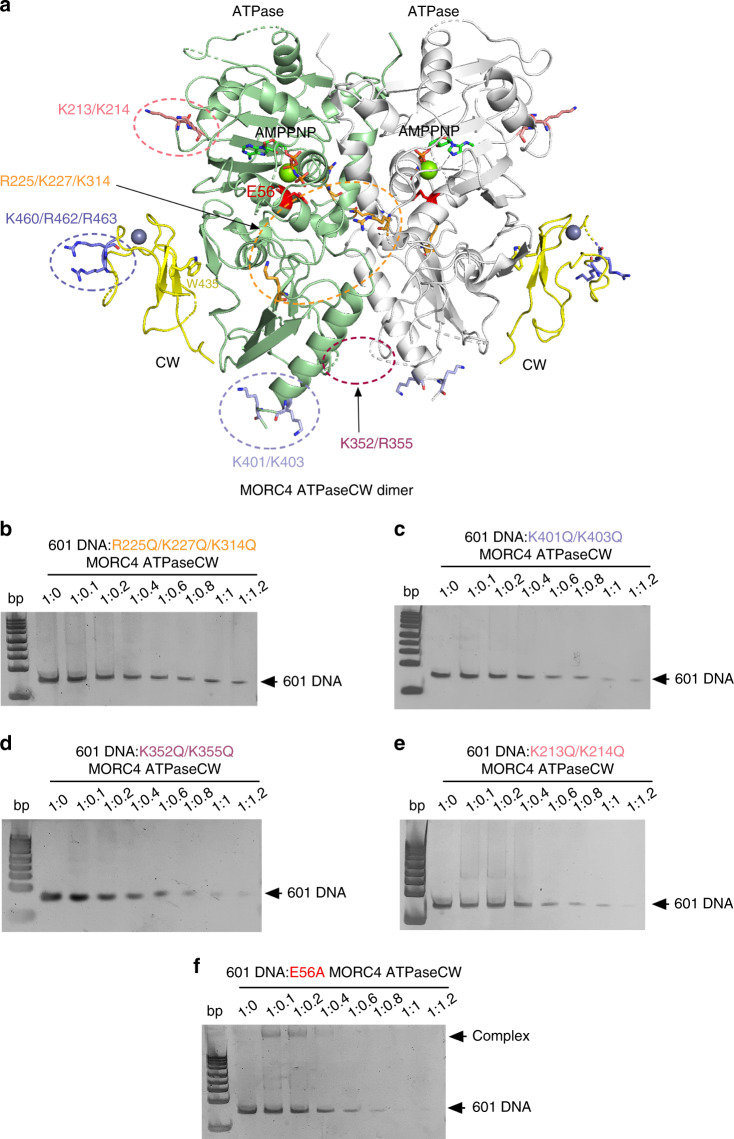
Mapping the DNA-binding site of the ATPaseCW cassette of MORC4. **a** A ribbon representation of the dimeric MORC4 ATPaseCW structure. The CW domain is colored yellow, and the ATPase domain is colored green in protomer A and gray in protomer B. The positively charged clusters in ATPaseCW are indicated by ovals and labeled. The residue E56, required for the catalytic activity of ATPaseCW, is also shown in red. **b**–**f** EMSA with 601 DNA in the presence of increasing amounts of indicated mutants of MORC4 ATPaseCW.

To delineate the DNA-binding site of the ATPaseCW cassette, we mutated four positively charged surface clusters in the ATPase domain, producing the K213Q/K214Q, R225Q/K227Q/K314Q, K352Q/R355Q, and K401Q/K403Q mutants of ATPaseCW, and tested binding of these mutants to 601 DNA by EMSA (Fig. 5). Among the mutants, the R225Q/K227Q/K314Q mutant (Fig. 5b), and to a lesser degree the K401Q/K403Q mutant (Fig. 5c), showed the most evident reduction in DNA-binding activity, whereas the reduction in DNA binding was less pronounced for other two mutants, K352Q/R355Q and K213Q/K214Q (Fig. 5d, e). The K460/R462/R463 cluster in the CW domain (Fig. 5a, blue oval) and the R225/K227/K314 and K401/K403 clusters in the ATPase domain (Fig. 5a, orange and light blue ovals) are separated by over 40 Å, indicating that a large surface of the ATPaseCW cassette is involved in binding to DNA. Furthermore, the R225/K227/K314 cluster is formed by R225 and K227 from one protomer and K314 from another protomer in the dimeric structure of ATPaseCW, suggesting that dimerization might be important for the association with DNA. Indeed, EMSA experiments showed that stimulation of ATPaseCW dimerization through adding AMPPNP increases binding to 601 DNA (Supplementary Figs. 5 and 6) and that at least ~30 bp DNA is needed for the ATPaseCW cassette to appreciably interact with DNA (Supplementary Fig. 7).

### MORC4 impedes binding of DNA-associated proteins

**Fig. 6:**
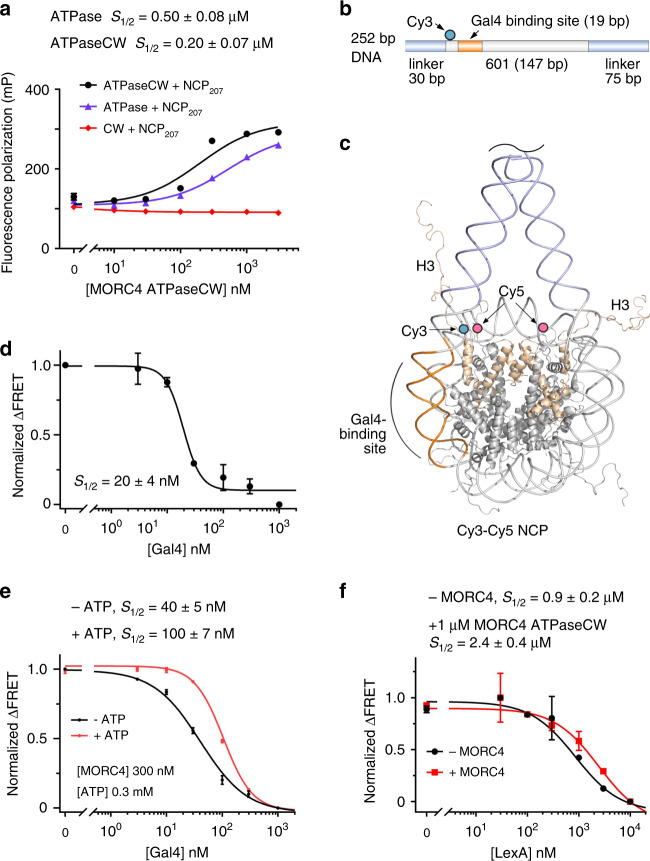
MORC4 ATPaseCW binds to and stabilizes nucleosomes. **a** Binding affinities and binding curves for the interactions of the indicated MORC4 regions with NCP_207_ as measured by fluorescence polarization. Data are represented as mean values +/− S.D. from three independent experiments (*n* = 3). **b** Schematic of the 252 bp DNA containing the 601 Widom sequence with a 75 bp linker at the 3′ end, a 30 bp linker at the 5′ end, and a Gal4 transcription factor binding site at bases 8–26 in the 601 Widom sequence with Cy3 positioned 34 bp from the 5′ end (cyan circle). **c** A model based on the crystal structure of the NCP with 197 bp palindromic 601 L DNA (PDB ID: 5NL0, the linker histone H1 is not shown) with histone H3 (wheat), Cy5 at H2AK119C (pink circles), Cy3 at the DNA (cyan circle), and the Gal4 target site (orange) are shown and labeled. Truncated DNA linkers are shown and colored light blue. **d**, **e** Normalized change in FRET efficiency of the Cy3-Cy5 labeled NCP_252_ upon addition of Gal4 in the absence (**d**) and presence (**e**) of MORC4 ATPaseCW and +/−ATP. Data are represented as mean values +/− S.D. from three independent experiments (*n* = 3). **f** Normalized change in FRET efficiency of the Cy3-Cy5 labeled NCP_147_ upon the addition of LexA in the absence and presence of MORC4 ATPaseCW. Data are represented as mean values +/− S.D. from three independent experiments (*n* = 3).

To gain insight into the MORC4 activity at chromatin, we investigated the consequences of the association of the MORC4 ATPaseCW cassette with NCP, a fundamental unit of chromatin. Both the ATPase domain alone and the ATPaseCW cassette of MORC4 bound to NCP_147_ (the nucleosome reconstituted with the 147 bp 601 nucleosome positioning DNA sequence) in fluorescence polarization assays (S_1/2_ = 2.3 µM and 0.4 µM, respectively) and EMSA (Supplementary Figs. 8 and 9). However, interaction of the ATPase domain and the ATPaseCW cassette with NCP_207_ (the nucleosome reconstituted with 207 bp DNA containing the 601 sequence flanked with a 30 bp linker on either side and internally labeled with fluorescein 27 bp in from the 5’ end) was increased ~5-fold and ~2-fold, respectively (Fig. 6a). These results suggest that extra-nucleosomal DNA enhances binding of MORC4. Importantly, binding of ATPaseCW to either NCP_147_ or NCP_207_ was ~2.5–6 fold tighter than the binding of the ATPase domain only, pointing to a contribution from the CW domain to the interaction with NCPs.

The impact of this interaction on the nucleosome dymnamics and unwrapping-wrapping equilibrium was investigated by Förster resonance energy transfer (FRET)^23^. We designed and prepared NCP_252_ using 252 bp DNA, which contained the 601 sequence with a 30 bp linker at the 5′ end, a 75 bp linker at the 3′ end, and the transcription factor Gal4 DNA-binding site replacing bases 8–26 in the 601 sequence (Fig. 6b). The Cy3 donor fluorophore was attached to the 5′ end of the 601 DNA adjacent to the Gal4-binding site, and the Cy5 acceptor fluorophore was attached to histone H2A(K119C). This placed Cy3 in the proximity of one of the Cy5 fluorophores, therefore a significant FRET signal is expected from a fully wrapped nucleosome, while a reduced FRET is expected when the NCP_252_ is in a more open, partially unwrapped state (Fig. 6c). Titration of the Gal4 DNA-binding domain into Cy3-Cy5 labeled NCP_252_ led to a decrease in FRET due to Gal4 binding to its target site and stabilization of the unwrapped state (Fig. 6d). Consistent with previous measurements^24^, the value for the Gal4 concentration at which FRET efficiency is reduced by 50% (S_1/2_) was found to be 20 nM.

To determine the effect of MORC4 on the nucleosome unwrapping-wrapping equilibrium, we next titrated Gal4 into NCP_252_ in the presence of ATPaseCW and ATP (Fig. 6e). The Gal4 ability to bind NCP_252_ was decreased ~5-fold, resulting in a S_1/2_ = 100 nM (Fig. 6e). The presence of ATP was essential, as only 2-fold decrease was observed in the absence of ATP (S_1/2_ = 40 nM). Similar results were obtained in FRET experiments using Cy3-Cy5 labeled NCP_147_ containing the targeting site of another transcription factor, LexA, at bases 8–27 in the 601 DNA sequence (Fig. 5f). Addition of MORC4 ATPaseCW to the reaction resulted in a decrease in binding of LexA to NCP_147_ due to the stabilization of the wrapped state of NCP_147_. Collectively, these data reveal that binding of the MORC4 ATPaseCW cassette to the nucleosome shifts the unwrapping-wrapping equilibrium toward the wrapped state, enhancing the nucleosome stability and impeding binding of DNA-binding proteins, such as transcription factors and co-activators.

### ATPase activity of MORC4 regulates formation of NBs

**Fig. 7:**
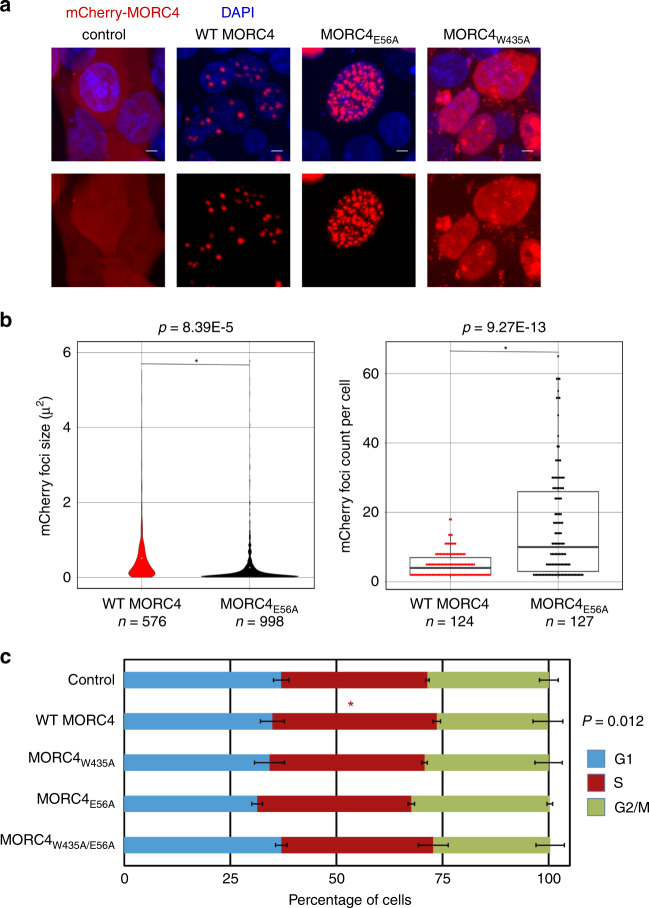
MORC4 forms NBs and affects the S phase cell cycle. **a** Representative confocal microscopy images of 293T-HEK cells overexpressing mCherry-CTRL, mCherry-MORC4 WT, mCherry-MORC4 E56A or mCherry-MORC4 W435A. Transfection were performed a minimum of three times, scale bars represent 5 μm. **b** MORC4 E56A shows a significant decrease in mCherry foci size (*p* = 8.39E−5) and a significant increase in number of mCherry foci per cell (*p* = 9.27E−13) compared to MORC4 WT. The analysis shown is from at least 10 independent images (WT MORC4 *n* = 13, MORC4 E56A *n* = 15), with a minimum of 120 nuclei analyzed for each condition (WT MORC4 *n* = 124, MORC4 E56A *n* = 127). **c** Cell cycle analysis following 48-h overexpression of MORC4 proteins exhibit increased percent of cells in S phase for WT MORC4 (*p* = 0.012) compared to control cells. Data represent the average of three independent experiments. Error bars represent S.E.M., * indicates significant difference from mCherry-CTRL (*p* < 0.05) by two-tailed student *t*-test.

Our findings demonstrate that the ATPaseCW cassette of MORC4 is capable of engaging chromatin. To determine the importance of this region in MORC4 functioning in vivo, we assessed the subcellular localization of MORC4 in HEK293T cells. Cells were transfected with mCherry-tagged wild-type and mutated full-length MORC4, and 48 h post transfection mCherry-tagged proteins were visualized in live cells by confocal fluorescence microscopy (Fig. 7a and Supplementary Fig. 10). We found that wild-type mCherry-MORC4 localizes to the nucleus, forming large NBs with the average size of 0.52 μm^2^. In 124 cells quantified, the number of MORC4 NBs ranges from 1 to 18 with an average of 4.93 per cell (Fig. 7b). The E56A mutant of MORC4 that retains its DNA-binding activity (Fig. 5f) but has catalytically impaired ATPase domain (Supplementary Fig. 11) showed a notable increase in the number of NBs and a decrease in the size of NBs in 127 cells quantified, indicating that the catalytic activity of MORC4 regulates the formation of NBs (Fig. 7a). In contrast, MORC4 W435A mutant, in which the structure of the CW domain is disrupted (Supplementary Fig. 12), was dispersed throughout the cell, revealing the critical role of CW in nuclear localization of MORC4 and its association with chromatin in vivo (Fig. 7a).

### MORC4 affects the S phase cell cycle

Chromatin organization is dynamic and undergoes extensive changes during the cell cycle, which requires chromatin condensation and decondensation. Because MORC4 enhances the nucleosome stability and homologous MORC1 was found to mediate chromatin compaction^25^, we tested whether MORC4 has a role in regulating the cell cycle. We assayed the cell cycle state distribution of ~10,000 HEK293T cells expressing mCherry-tagged wild-type and mutant MORC4 by flow cytometry (Fig. 7c and Supplementary Fig. 13). We found that cells expressing wild-type MORC4 showed a modest, but significantly larger population in S phase of the cell cycle (38.7%) compared with the control cells (34.5%). These results indicate that overexpression of MORC4 alters S phase progression in HEK293T cells. Notably, neither the ATPase mutant (E56A, 36.3%) nor the CW mutant (W435A, 36.5%) or the dual mutant (E56A/W435A, 35.8%) altered S phase progression to the same degree. These data reveal that both ATPase activity and the intact CW domain are essential (Fig. 7c), but no significant effect was observed for the K460A/R462A/R463A mutant, which suggested that the disruption of the DNA-binding activity of CW is not enough (Supplementary Fig. 13). Our results are consistent with a model whereby MORC4 stabilizes the nucleosome resulting in decreased chromatin accessibility during S phase and slowing replication of DNA. Together, our data point to a role of MORC4 in S-phase progression, which depends on both its catalytic activity and the CW domain.

## Discussion

Among four members of the MORC family of proteins, MORC4 is the least well characterized. In this study we report the molecular and structural mechanism underlying the catalytic ATPase activity of MORC4. Our current model for the MORC4 activation suggests that binding of the ATPase domain and the CW domain of the ATPaseCW cassette to DNA stimulates the catalytic activity of MORC4 and promotes binding of CW to H3K4me3 (Fig. 4b). Notably, our data demonstrate that the DNA-binding function of CW is required for the MORC4 activation, as the mutant protein with the impaired DNA-binding site of CW loses its ability to be stimulated to hydrolyze ATP.

Despite the overall similar architecture of the MORC proteins, their mechanisms for enzymatic activation differ markedly. In contrast to MORC4, the CW domain of MORC3 does not bind DNA and its coupling to the ATPase domain prevents DNA binding by the ATPase domain^13,15^. This results in an autoinhibited, catalytically inactive state of MORC3, which is released through the interaction of CW with histone H3K4me3. In contrast to MORC4 and MORC3, it has been shown that following DNA damage, MORC2 is phosphorylated on Ser739, and this phosphorylation is required for the ATPase activity of MORC2^26^. The distinctive mechanisms of action of MORCs correlate with the fact that these proteins have unique DNA-binding regions: (i) encompassing the ATPase and CW domains in MORC4, (ii) the ATPase domain only, with the CW domain impeding binding to DNA in MORC3^13,15^, and (iii) a coiled coil (CC) insertion between the ATPase domain and the CW domain in MORC2^17,27^. While both DNA binding and dimerization are essential for the ATPase activity of MORC3, mutation of the MORC2 residues that disrupt dimerization interface raises the rate of ATP hydrolysis, and this observation led to a model of MORC2 cycling between monomeric and dimeric states^17^.

Furthermore, MORCs are characterized by different chromatin engaging modes, as their CW domains have dissimilar functions. MORC2 contains a CW domain that is lacking one of the aromatic cage tryptophan residues and thus does not recognize histones. The CW domain of MORC3 is incapable of binding to DNA, and its histone-binding function mediates both the chromatin association and ATPase activity of MORC3. Unlike MORC3 CW, the CW domain of MORC4 is essential in chromatin targeting and binds to histones but it is its DNA-binding function that is necessary for the ATPase activity of MORC4. The CW domain does not inhibit the DNA-binding activity of MORC4 and MORC2, whereas the CW domain of MORC3 does.

Given the stark differences in the mechanisms of action of MORCs, it is expected that each MORC family member may be involved in distinct biological events. Here, we show that in cells, MORC4 plays a role in S-phase progression, possibly by decreasing chromatin accessibility during S phase and slowing replication of DNA, as in vitro results demonstrate that MORC4 promotes the DNA wrapping in the nucleosome in FRET experiments. While MORC2 is found in the nuclear and cytosolic pools, MORC4 and MORC3 localize exclusively to the nucleus where they form NBs. We have previously shown that MORC3 NBs have LLPS gel-like properties^20^, and it will be essential in future studies to determine the nature of MORC4 NBs. Furthermore, to better understand their roles in chromatin condensation or decondensation, it will be imperative to elucidate the structural modes of MORCs’ engagement with the nucleosome.

## Methods

### Protein expression and purification

The ATPase domain (aa 29–411) and the ATPaseCW cassette (aa 29–486) of human MORC4 (Q8TE76) were cloned into a pDEST17 vector and pDEST15 vector, respectively. The CW domain (aa 417–475) of MORC4 was cloned into a pGEX 6p-1 vector. Proteins were expressed in Rosetta2 (DE3) pLysS or BL21 (DE3) RIL in LB or minimal media supplemented with ^15^NH_4_Cl. Protein expression was induced with 0.2 mM IPTG for 16 h at 16 °C. The His-tagged ATPase domain was purified on HisPur Ni-NTA agarose beads (Thermo-Fisher) in 20 mM Tris-HCl (pH 7.5), 300 mM NaCl, 5 mM MgCl_2,_ and 2 mM DTT. The GST-tagged ATPaseCW cassette was purified on Pierce glutathione agarose beads (Thermo-Fisher) in 20 mM Tris-HCl (pH 7.5), 300 mM NaCl, 5 mM MgCl_2,_ and 2 mM DTT. GST-CW was purified on Pierce glutathione agarose beads (Thermo-Fisher) in 20 mM Tris-HCl (pH 7.5) buffer supplemented with 150 mM NaCl and 2 mM DTT. The GST tag was cleaved overnight at 4 °C with either TEV or PreScission proteases. Unlabeled proteins were further purified by size exclusion chromatography and concentrated in Millipore concentrators. All mutants were generated by site-directed mutagenesis using the Stratagene QuikChange mutagenesis protocol, grown and purified as wild-type proteins. MORC3 CW was expressed and purified as reported^15^.

### X-ray crystallography

The human MORC4 ATPaseCW cassette (aa 29–486) solution was concentrated to 6 mg/mL and incubated with the H3K4me3 peptide (aa 1–12 of H3, in a 1:2 protein-to-peptide molar ratio) along with 2 mM AMPPNP at 25 °C for 20 min in buffer containing 20 mM Tris-HCl (pH 7.5), 5 mM MgCl_2_ and 2 mM DTT. Crystals were obtained at 18 °C using the sitting-drop vapor diffusion method by mixing equal volumes of protein solution with well solution composed of 0.1 M Hepes (pH 7.5) and 20% Jeffamine ED-2003. X-ray diffraction data were collected from a single crystal at the ALS 4.2.2 beamline, Berkeley administrated by the Molecular Biology Consortium. HKL2000 was used for indexing, scaling, and data reduction. The phase solution was obtained by molecular replacement using the MORC3 ATPaseCW structure (PDB ID 6O1E) as a search model. Manual model building was performed using Coot^28^, and the structure was refined using Phenix^29^. The final structure was verified by MOLProbity^30^ and the PDB validation server. The X-ray diffraction and structure refinement statistics are summarized in Supplementary Table 1.

### Fluorescence spectroscopy

Spectra were recorded at 25 °C on a Fluoromax-3 spectrofluorometer (HORIBA). The samples containing 0.5–1 µM wild-type or mutated ATPaseCW or CW in 20 mM Tris pH 6.8, 100 mM NaCl, 2 mM DTT buffer and progressively increasing concentrations of the histone peptides were excited at 295 nm. Emission spectra were recorded between 320 and 360 nm with a 0.5 nm step size and a 0.5 s integration time and averaged over three scans. The *K*_d_ values were determined using a nonlinear least-squares analysis and the equation:

1 $$\Delta I = \Delta {I}_{\mathrm{max}}\frac{{\left( {\left( {\left[ L \right] + \left[ P \right] + K_{\mathrm{d}}} \right) - \sqrt {\left( {\left[ L \right] + \left[ P \right] + K_{\mathrm{d}}} \right)^2 \,-\, \left. {4\left[ P \right]\left[ L \right]} \right)} } \right)}}{{2\left[ P \right]}}$$

where [*L*] is the concentration of the histone peptide, [*P*] is the concentration of the protein, Δ*I* is the observed change of signal intensity, and Δ*I*_max_ is the difference in signal intensity of the free and bound states of the protein. The *K*_d_ values were averaged over three separate experiments with the error calculated as the standard deviation between the runs.

### ATPase activity assays

The ATPase assays were performed using the EnzChek Phosphate Assay Kit (Invitrogen, item # E6646). The reactions were carried out on 1.0 µM of MORC4 His-ATPase or ATPaseCW (WT and mutants), in the presence and absence of 1 µM 601 DNA or 50 µM of unmodified H3 or H3K4me3 peptide (aa 1–12 of H3) in a buffer containing 50 mM Tris-HCl (pH 7.5), 150 mM NaCl, 1 mM MgCl_2_, 0.1 mM sodium azide, 200 µM MESG, and 1 U of PNP. In assay with the nucleosome, 0.5 µM of MORC4 ATPaseCW and 0.5 µM NCP_167_ (Activemotif) were used. The reaction was started by adding 2 mM ATP to the mixture at room temperature, and the release of inorganic phosphate was monitored by measuring the absorbance at 360 nm on a Nanodrop 2000c spectrophotometer (Thermo Scientific). In the presence of inorganic phosphate, produced by the hydrolysis of ATP to ADP, MESG is enzymatically converted to ribose 1-phosphate and MESG by PNP, resulting in a shift in the wavelength absorbance from 330 nm for MESG to 360 nm for the product. The rate of ATP self-hydrolysis was measured in parallel. Error was calculated as the S.D. of at least three separate experiments (two in Fig. 4j).

### NMR experiments

NMR experiments were carried out at 298 K on a Varian INOVA 600 and 900 MHz spectrometers. His-tagged MORC4 ATPase (1–411) was expressed and purified as described above and used without cleavage of the tag. NMR samples contained 0.1 mM uniformly ^15^N-labeled WT or mutated CW in 20 mM Tris-HCl (pH 7) buffer supplemented with 100 mM NaCl, 5 mM DTT, and 10% D_2_O. Binding was characterized by monitoring CSPs in the proteins induced by H3 peptides or the ATPase domain. For the CW_KRR_ mutant, NMR experiments were performed in 20 mM Tris-HCl (pH 6.8) buffer supplemented with 150 mM NaCl, 2 mM DTT, and 10% D_2_O.

### Peptide microarrays

Histone peptide microarrays were produced, performed, and analyzed as described^31^ with some modifications. Microarrays were analyzed by the Typhoon Trio+ (GE) and quantified using the ImageQuant TL software. The analysis was performed by first averaging the triplicate values for each peptide in a subarray; these were then linearly scaled based on the minimum and maximum values to be between 0 and 1. These scaled values were then averaged together and sorted based on the modifications stated in the plot legends. Averages and standard deviations are from ≥3 arrays; these values for every peptide tested can be found in Supplementary Data 1.

### Histone peptide pulldown assays

The in-solution histone peptide pulldowns experiments were performed as described^31^. In brief, 50 pmol of MORC4 CW was incubated with 500 pmol of each biotinylated histone peptide in 50 mM Tris, 0.1% NP-40, 0.5% BSA, 500 mM NaCl, pH 8.0 buffer for one hour at 4 °C with rotation. Five microliters of magnetic streptavidin-coated beads were added to each reaction and the mixtures were incubated for another hour with rotation. The beads were then washed three times and analyzed using SDS–PAGE and western blotting. Anti-GST (13-0022, 1:1000) antibody was from EpiCypher and anti-Rabbit-HRP (NA934V, 1:20,000) antibody was from GE. The input control lane represents 1% of the input material used in a pulldown. The images shown are representative of three experiments.

### EMSA with DNA

EMSA experiments were performed essentially as described^32^. In brief, increasing amounts of WT or mutant CW or ATPaseCW cassette of MORC4 were incubated with 147 bp 601 DNA (5 pmol) or 50 ng of O’RangeRuler 5 bp ladder (Thermo Scientific) in a DNA-binding buffer containing 20 mM Tris-HCl (pH 7.5), 150 mM NaCl, 2.5 mM MgCl_2_, and 2 mM DTT for 0.5 h on ice. The reaction mixtures were loaded on 5% native polyacrylamide gels, and electrophoresis was performed in 0.2× Tris-borate-EDTA (TBE) buffer at 100 V for 1.2 h on ice. Gels were stained with SYBR Gold (Invitrogen).

### Cross-linking assay

His-tagged MORC4 (aa 1–486) and MORC4 (29–283, I30A) were expressed and purified as described above. 10 μM proteins were cross-linked in 10 mM Hepes pH 7.5, 150 mM NaCl, 2 mM MgCl_2_, 0.5 mM TCEP, and 1 mM AMPPNP or ADP. BS3 cross-linker (Thermo) was resuspended to 2 mM and used at a concentration of 0.1 mM. Cross-linking was allowed to proceed for 10 min. Cross-linked samples were quenched in 0.1 M Tris pH 7.5, resolved on SDS/PAGE, and stained with Coomassie blue.

### Fluorescence polarization

Fluorescence polarization measurements were acquired with a Tecan infinite M1000Pro plate reader by exciting at 470 nm and measuring polarized emission at 519 nm with 5 nm excitation and emission bandwidths. The fluorescence polarization was calculated from the emission polarized parallel and perpendicular to the polarized excitation light. Fluorescence polarization measurements were carried out using increasing concentrations of MORC4 ATPaseCW, ATPase alone, or CW alone with 5 nM 207 bp Fluorescein-labeled NCPs in 15 mM Tris pH 7.5, 75 mM NaCl, 0.00625% Tween 20, and 5 mM dithiothreitol buffer in 30 µL reaction volumes. The samples were loaded into a Corning round bottom black polystyrene plate and incubated at room temperature for 10 min before fluorescence polarization measurements of the samples were taken. The data were then fit to a non-cooperative binding isotherm to determine S_1/2_ values. Errors represent a S.D. between the S_1/2_ values based on three runs.

### NCP preparation for fluorescence polarization and FRET

Histone octamers unlabeled or labeled with Cy5 at H2AK119 were prepared as previously described^33^. NCPs were reconstituted by combining octamer with 1.25× excess DNA and performing slow salt dialysis. Free DNA was then separated from fully reconstituted NCPs by sucrose gradient purification. NCPs for fluorescence polarization assays were reconstituted from unlabeled octamer and 207 bp DNA containing the 601 Widom sequence flanked with a 30 bp linker on either side and internally labeled with Fluorescein 27 bp in from the 5′ end. Cy3-Cy5 labeled NCPs for FRET assays were reconstituted with Cy5 labeled octamer plus either 147 bp 601 Widom DNA containing a LexA transcription factor binding site at bases 8–27^34^, or 252 bp DNA containing the 601 Widom sequence with a 75 bp linker at the 3′ end, a 30 bp linker at the 5’ end, and a Gal4 transcription factor binding site at bases 8–26 in the 601 Widom sequence. The 147 bp LexA-binding DNA was labeled with Cy3 on the 5′ end, and the 252 bp Gal4-binding DNA was internally labeled with Cy3 34 bp in from the 5′ end.

### LexA and Gal4 protein preparation

LexA was expressed in *E*. *coli*, separated from genomic DNA and the proteome by polyethyleneimine precipitation, and salted out with ammonium sulfate. LexA was resuspended in buffer A (20 mM potassium phosphate pH 7, 0.1 mM EDTA, 10% glycerol, 1 mM DTT) + 200 mM NaCl and purified by a linear gradient to A + 800 mM NaCl over either a cellulose phosphate or HiTrap Heparin HP column. Final LexA purification was performed on a hydroxyapatite column and dialyzed into 10 mM PIPES pH 7.0, 0.1 mM EDTA, 10% glycerol, and 200 mM NaCl for storage at −80 °C. The His-tagged DNA-binding domain (aa 1–147) of Gal4 was expressed in *E*. *coli* and purified from lysate by a linear gradient of buffer B (25 mM Tris pH 7.5, 200 mM NaCl, 0.2% Tween 20, 20 µM zinc acetate, 1 mM dithiothreitol, and 1 mM PMSF) + 10 mM imidazole to B + 200 mM imidazole over a Nickel-NTA column. Gal4 (1–147) was then further purified by a linear gradient of buffer C (25 mM Tris pH 7.5, 20 µM zinc acetate, 1 mM dithiothreitol, and 1 mM PMSF) + 200 mM NaCl to C + 800 mM NaCl over a Tosoh cation exchange column. Purified Gal4 (1–147) was then dialyzed into 10 mM HEPES pH 7.5, 200 mM NaCl, 10% glycerol, 20 µM zinc acetate, 1 mM DTT, and 1 mM PMSF for storage at −80 °C.

### FRET

Cy3-Cy5 NCP FRET efficiency measurements were carried out on a Horiba Scientific Fluoromax 4. Samples were excited at 510 and 610 nm and the photoluminescence spectra were collected from 530 to 750 nm and 630 to 750 nm for donor and acceptor excitations, respectively. Each wavelength was integrated for one second, and the excitation and emission slit width were set to 5 nm with 2 nm emission wavelength steps. FRET measurements were computed through the (ratio)A method. LexA titrations were carried out in 20 mM Tris pH 7.5, 75 mM NaCl, 10% glycerol, 0.00625% Tween 20 buffer with 5 nM 147 bp Cy3-Cy5 NCPs mixed in 20 µL reaction volumes and allowed to incubate for 5 mins at 4 °C before FRET of the sample was measured. To measure changes in accessibility of the NCP_147_ due to MORC4 ATPaseCW binding, LexA titrations were repeated in the presence of NCP binding saturating concentrations of MORC4 ATPaseCW. Gal4 (1–147) titrations were carried out in 10 mM Tris pH 8, 130 mM NaCl, 10% glycerol, 0.0075% Tween 20 buffer with 1 nM 252 bp Cy3-Cy5 nucleosomes mixed in 60 µL reaction volumes and allowed to incubate for 10 mins at room temperature before FRET of the sample was measured. To measure changes in accessibility of the NCP_252_ due to MORC4 ATPaseCW binding, Gal4 (1–147) titrations were repeated in the presence of NCP binding saturating concentrations of MORC4 ATPaseCW +/− 0.3 mM ATP. FRET efficiency values were normalized against the FRET efficiency value of NCP in the absence of titrant. The titrations were then fit to a non-cooperative binding isotherm to determine S_1/2_ values. Errors represent a S.D. between the S_1/2_ values based on three runs.

### EMSA with NCPs

EMSAs were performed by mixing increasing amounts of MORC4 ATPase with 5 nM 147 bp fluorescein-labeled NCP in 25 mM Tris-HCl pH 7.5, 75 mM NaCl, 25 mM Tris-HCl pH 7.5, 0.005% Tween 20, and 10% glycerol buffer in a 12 μL reaction volume. Each sample was incubated at 4 °C for 5 min and then loaded onto a 5% native polyacrylamide gel. Electrophoresis was performed in 0.3× Tris-borate-EDTA (TBE) at 300 V for 90 min. Fluorescein fluorescence images were acquired with a Typhoon Phosphor Imager.

### Cell culture

293T-HEK cells were maintained in Dulbecco’s Modified Essential Medium (DMEM) with 10% FB Essence (VWR), 1% penicillin/streptomycin and L-glutamine.

### MORC4 localization in HEK cells

WT mCherry-MORC4, mCherry-MORC4 E56A, mCherry-MORC4 W435A, mCherry-MORC4 E56A/W435A, mCherry-MORC4 K460A/R462A/R463A, or mCherry control plasmid were transfected into 2 million 293T-HEK cells seeded on 25 mm circular coverslips (Carolina Biological Supply Item#: 633057) in 10-cm-diameter tissue culture dish by Lipofectamine 3000 (Life Technology, L3000-075) following the manufacturer’s instructions. Two days after transfection, cells were stained with Hoechst 333241 for 30 min and imaged live on a ZeissAxiovert 200 M inverted microscope fitted with a ×40 oil objective. Images were acquired with Slidebook 6 software and exported for analysis in Image J.

### mCherry foci size and foci per cell calculations

mCherry foci sizes were calculated using ImageJ image processing program (version 1.51) using the Analyze Particle tool. mCherry particle sizes were collected for MORC4 WT and MORC4 W435A transfected cells. Data were plotted with R (version 3.4.1) using ggplot2 (version 3.2.1). Cell size and mCherry foci count per cell were calculated using CellProfile cell image analysis software (version 3.1.5) to identify nuclei and mCherry foci, relate mCherry foci to nuclei containing them and measure the size of nuclei and number mCherry foci contained. Data were plotted with R (version 3.4.1) using ggplot2 (version 3.2.1).

### Flow cytometry

For DNA content analysis, 10^6^ cells previously transfected with mCherry MORC4 constructs and labeled with 25 µM Ethynyl deoxyUridine (EdU) for 1 h in 100 µL were washed with PBS (NaCl 137 mM, KCl 2.7 mM, Na_2_HPO_4_ 10 mM, KH_2_PO_4_ 1.8 mM) and fixed in ice cold 70% ethanol overnight. Fixed cells were washed and resuspended in PBS containing 0.05% NP40 and incubated at room temperature for 30 min. Cells were again washed with PBS. Cells were resuspended in Alexa Fluor 647 click staining solution (100 mM Tris pH 7.6, 4 mM CuSO_4_, 3 µM AF647 azide, 100 mM Sodium Ascorbate) for 1 h. Following staining, cells were washed with PBS and then stained for total DNA content using 10 μg/mL propidium iodide (PI) and 0.2 mg/ml RNase A for 60 min before analysis on a FACScan instrument. Data were analyzed using FlowJo (Version 10) with cells gated to remove doublets. EdU vs PI plots were gated in quadrants to identify G1, early S, late S and G2/M cells. S phase fractions were summed for total S phase population. Four biological replicated were conducted and the average percentages for each phase were plotted.

### Reporting summary

Further information on research design is available in the Nature Research Reporting Summary linked to this article.

## Supplementary information

Supplementary Information

Peer Review File

Reporting Summary

Description of Additional Supplementary Files

Supplementary Data 1

## Data Availability

Coordinates and structure factors for the ATPaseCW cassette have been deposited in the Protein Data Bank (PDB 7K7T). All relevant data supporting the key findings of this study are available within the article and its Supplementary Information files or from the corresponding author upon reasonable request. Source data are provided with this paper.

## Source data

Source Data
