# Supplementary material for: Molecular mechanism of the MORC4 ATPase activation
Source: Nat Commun. 2020 Oct 29;11:5466. doi: 10.1038/s41467-020-19278-8 (PMC7596504; doi:10.1038/s41467-020-19278-8)
Supplement: Supplementary file 1 — Supplementary Information [file 41467_2020_19278_MOESM1_ESM.pdf]

Supplementary Information

Molecular mechanism of the MORC4 ATPase activation

Adam H. Tencer[1], Khan L. Cox[2], Gregory M. Wright[1], Yi Zhang[1], Christopher J. Petell[3], Brianna J. Klein[1], Brian D. Strahl[3], Joshua C. Black[1], Michael G. Poirier[2], and Tatiana G. Kutateladze[1]*

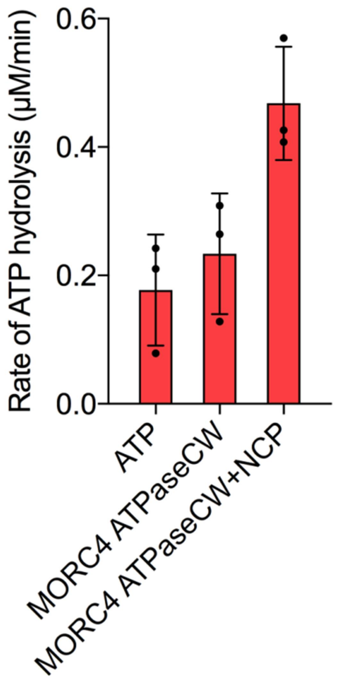

Supplementary Figure 1. Rates of ATP hydrolysis by the ATPase-CW cassette of MORC4 in the presence and absence of NCP_{167} (the nucleosome containing 167 bp DNA). Data are represented as mean values +/- S.D. from three independent experiments (n=3). Source data are provided in a Source Data file.

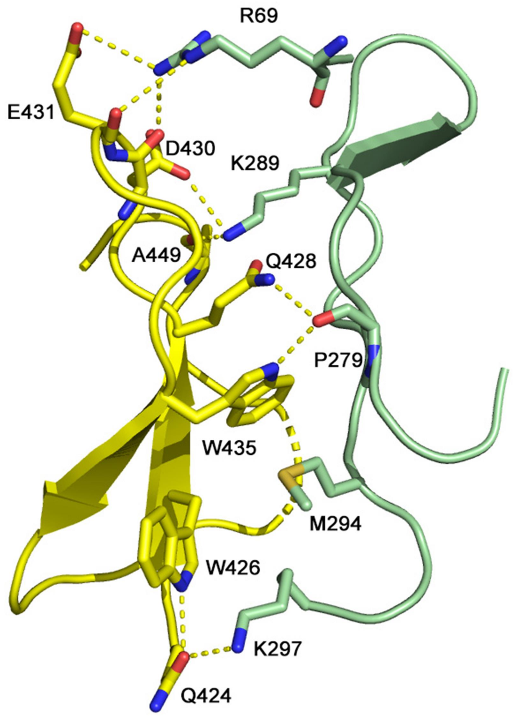

Supplementary Figure 2. A zoom-in view of the ATPase:CW interface from the structure of the ATPaseCW cassette. Dashed lines indicate hydrogen bonds between the ATPase domain residues (green) and the CW domain residues (yellow).

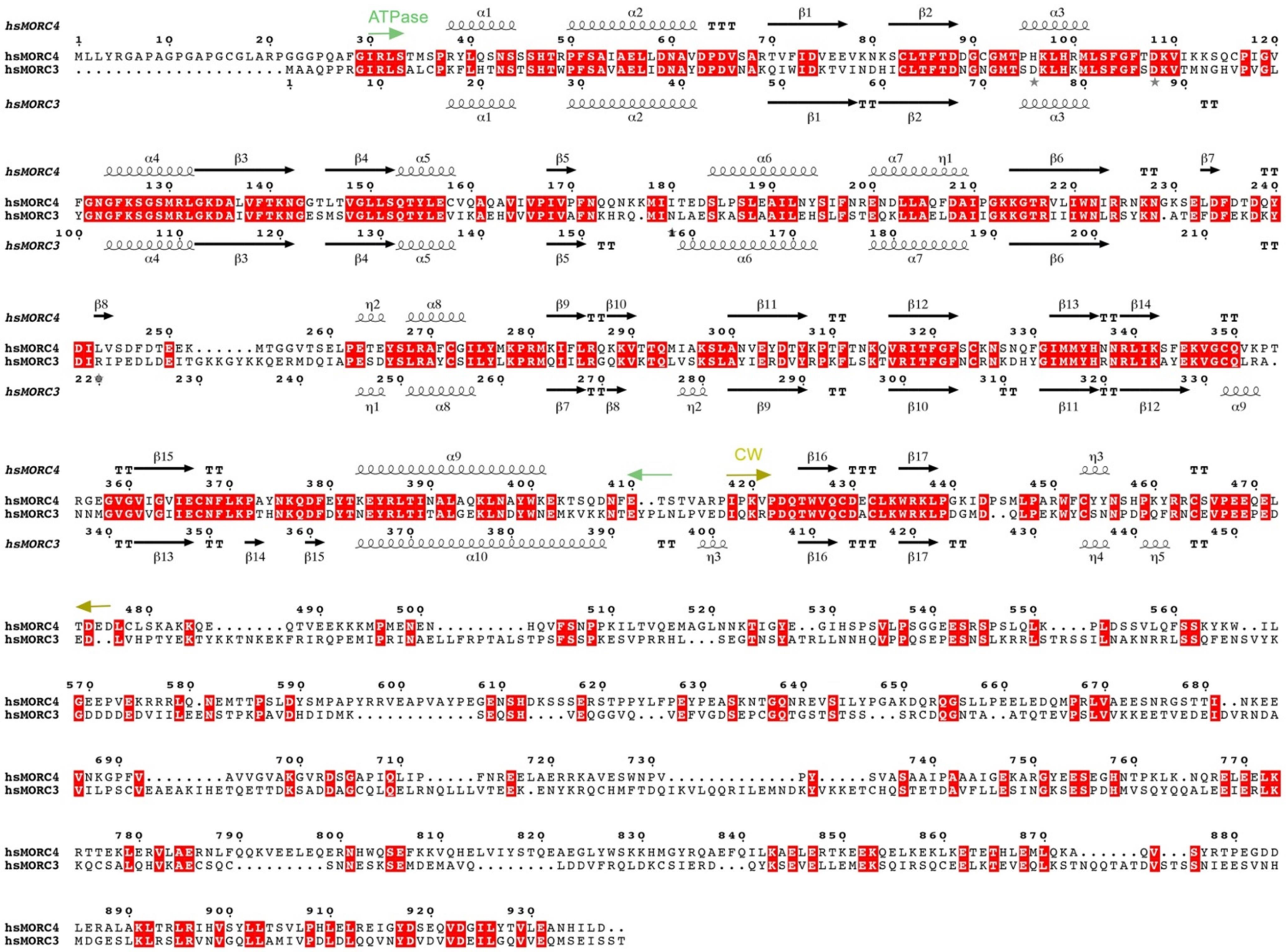

Supplementary Figure 3. Alignment of the amino acid sequences from MORC4 and MORC3. Identical residues are highlighted red. The ATPase and CW domains are indicated by green and yellow arrows and labeled.

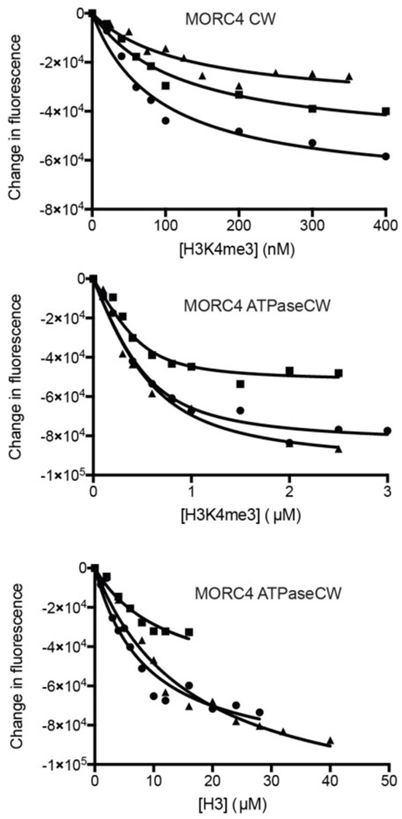

Supplementary Figure 4. Binding curves used to determine the K_d values by tryptophan fluorescence.

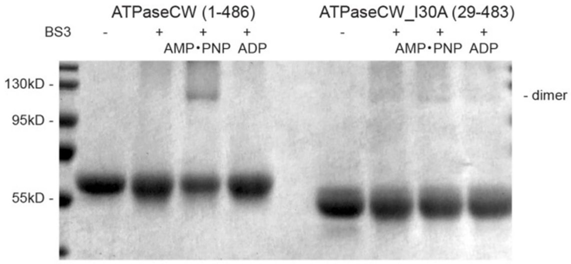

Supplementary Figure 5. Cross-linking assays using WT MORC4 ATPaseCW and I30A mutant of MORC4 ATPaseCW (designed based on sequence alignment with MORC3 to abolish dimerization) in the absence or presence of AMPPNP or ADP. AMPPNP but not ADP induced dimerization. Experiment with ATPaseCW (1-486) was repeated two times.

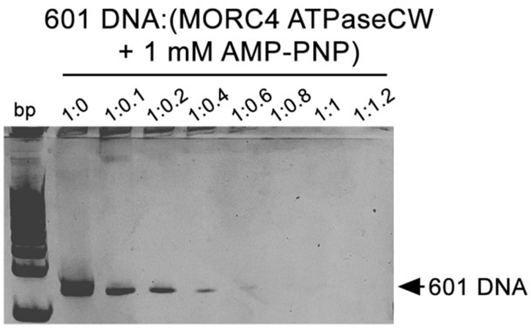

Supplementary Figure 6. EMSA with 601 DNA in the presence of 1 mM AMPPNP and increasing amounts of WT MORC4 ATPaseCW.

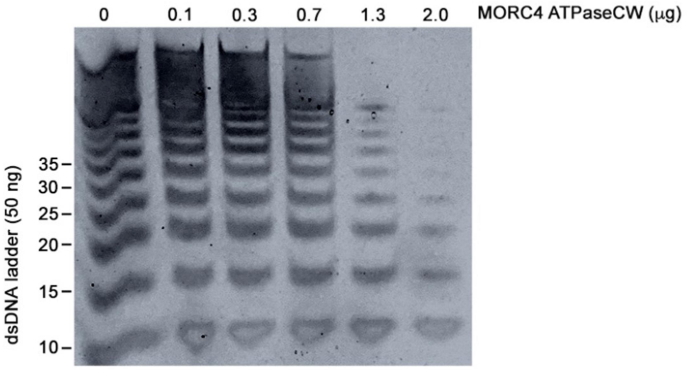

Supplementary Figure 7. EMSA with 5bp dsDNA ladder (50 ng) and increasing amounts of WT MORC4 ATPaseCW, as indicated above the gel. Experiment was repeated three times.

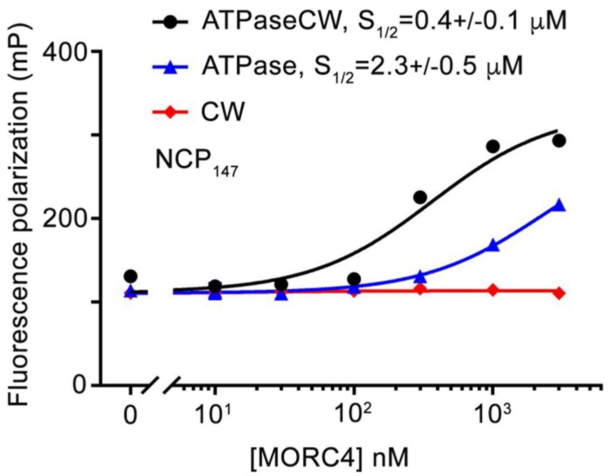

Supplementary Figure 8. Binding affinities and binding curves for the interactions of the indicated MORC4 regions with NCP_{147} as measured by fluorescence polarization. Data are represented as mean values +/- S.D. from three independent experiments (n=3).

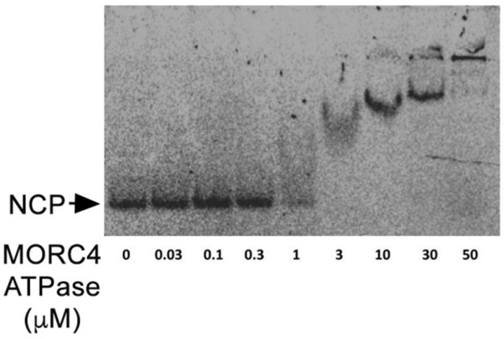

Supplementary Figure 9. EMSA with NCP_{147} in the presence of increasing amounts of the ATPase domain of MORC4. Experiment was repeated three times.

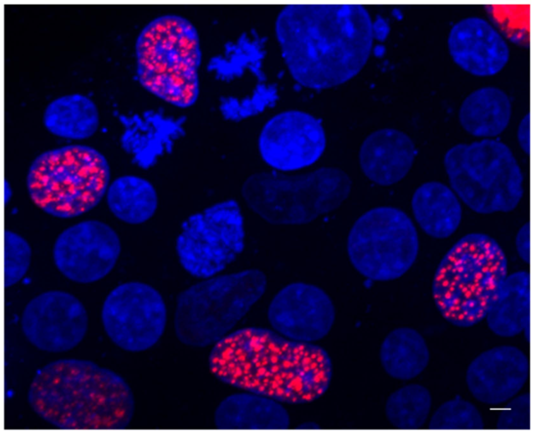

Supplementary Figure 10. Representative confocal microscopy images of 293T-HEK cells overexpressing mCherry-MORC4 E56A. Transfection were performed a minimum of 3 times, scale bar represents 5 μm.

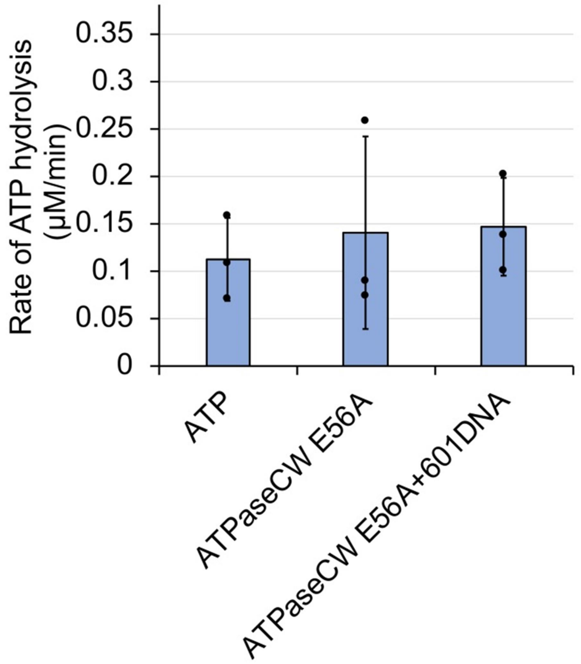

Supplementary Figure 11. The E56A mutant of MORC4 is catalytically inactive but binds DNA as WT MORC4. Rates of ATP hydrolysis by the E56A mutant of the ATPaseCW cassette of MORC4 in the presence and absence of 601 DNA. Data are represented as mean values +/- S.D. from three independent experiments (n=3). Source data are provided in a Source Data file.

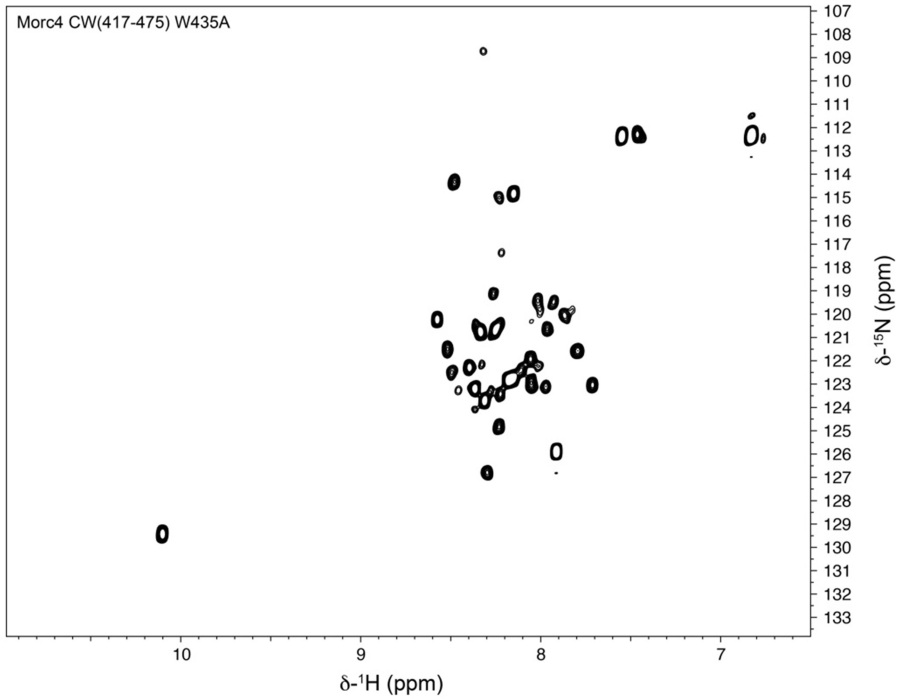

Supplementary Figure 12. $^{1}H,^{15}N$ HSQC spectrum of the ^{15}N-labeled MORC4 CW_{W435A} mutant indicates an unfolded protein.

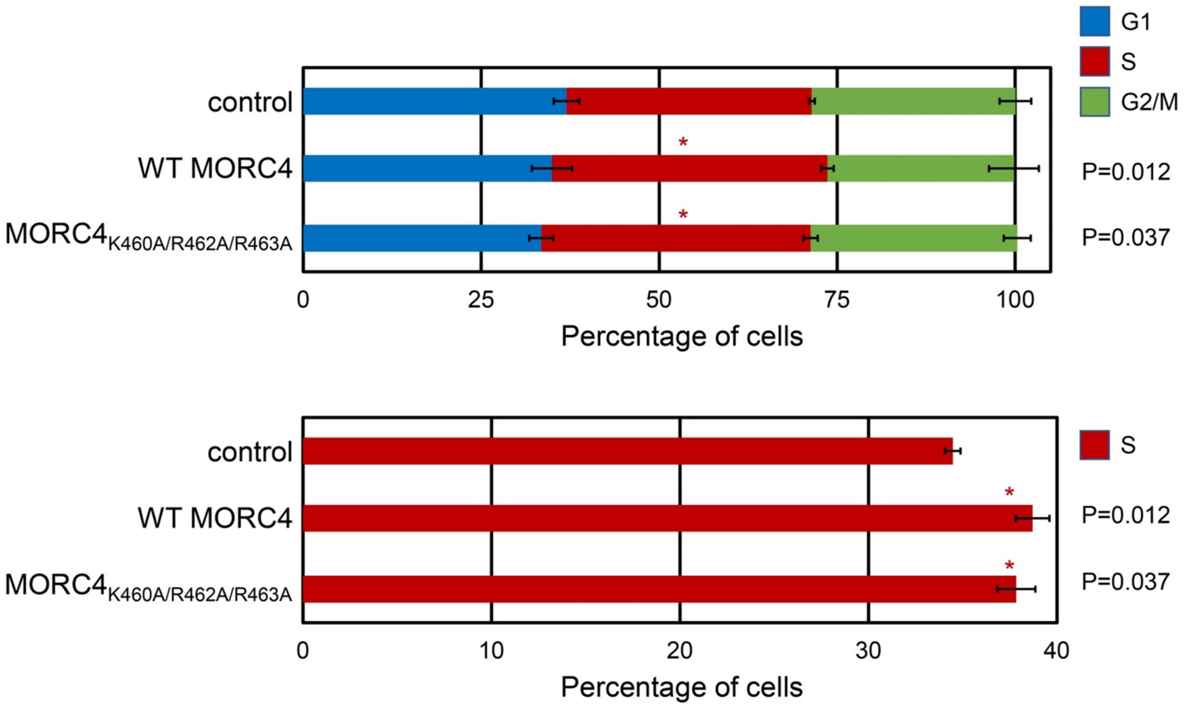

Supplementary Figure 13. Cell cycle analysis following 48 hour overexpression of MORC4 proteins exhibit increased percent of cells in S phase for WT MORC4 (p=0.012) and MORC4 K460A/R462A/R463A mutant (p=0.037) compared to control cells. Data represent the average of three independent experiments. Error bars represent S.E.M, * indicates significant difference from mCherry-CTRL (p<0.05) by two-tailed student t-test.

Supplementary Table 1. Data collection and refinement statistics

	MORC4 ATPaseCW/AMPPN
**Data collection**	
Space group	P 1 21 1
Cell dimensions	
a, *b*, *c* (Å)	52.4, 109.9, 70.4
α, β, γ (°)	90.0, 96.0, 90.0
Resolution (Å)	2.9(3.1-2.9) *
R_{pim}	7.9(28.5)
$I / \sigma I$	10.1(2.0)
Completeness (%)	94.7(75.5)
Redundancy	3.5(2.4)
**Refinement**	
Resolution (Å)	47.1-2.9
No. reflections	15106
R_{work} / R_{free}	0.2397/0.2644
No. atoms	6338
ATPaseCW	6242
AMPPNP/Mg/Zn	66
Water	30
B-factors	39.66
ATPaseCW	39.76
AMPPNP/Mg/Zn	32.17
Water	34.64
R.m.s. deviations	
Bond lengths (Å)	0.003
Bond angles (°)	0.588
Ramachandran plot	95.92
Most favored (%)	3.94
Allowed (%)	0.14
Outliers (%)	

*Values in parentheses are for highest-resolution shell.
