# Supplementary material for: Molecular mechanism of the MORC4 ATPase activation
Source: Nat Commun. 2020 Oct 29;11:5466. doi: 10.1038/s41467-020-19278-8 (PMC7596504; doi:10.1038/s41467-020-19278-8)
Supplement: Supplementary file 3 — Reporting Summary [file 41467_2020_19278_MOESM3_ESM.pdf]

nature research

Corresponding author(s): Tatiana Kutateladze

Reporting Summary

Nature Research wishes to improve the reproducibility of the work that we publish. This form provides structure for consistency and transparency in reporting. For further information on Nature Research policies, see Authors & Referees and the Editorial Policy Checklist.

Statistical parameters

When statistical analyses are reported, confirm that the following items are present in the relevant location (e.g. figure legend, table legend, main text, or Methods section).

n/a	Confirmed	
	☒	The exact sample size (*n*) for each experimental group/condition, given as a discrete number and unit of measurement
	☒	An indication of whether measurements were taken from distinct samples or whether the same sample was measured repeatedly
☒		The statistical test(s) used AND whether they are one- or two-sided<br>*Only common tests should be described solely by name; describe more complex techniques in the Methods section.*
☒		A description of all covariates tested
☒		A description of any assumptions or corrections, such as tests of normality and adjustment for multiple comparisons
	☒	A full description of the statistics including central tendency (e.g. means) or other basic estimates (e.g. regression coefficient) AND variation (e.g. standard deviation) or associated estimates of uncertainty (e.g. confidence intervals)
☒		For null hypothesis testing, the test statistic (e.g. *F*, *t*, *r*) with confidence intervals, effect sizes, degrees of freedom and *P* value noted<br>*Give P values as exact values whenever suitable.*
☒		For Bayesian analysis, information on the choice of priors and Markov chain Monte Carlo settings
☒		For hierarchical and complex designs, identification of the appropriate level for tests and full reporting of outcomes
☒		Estimates of effect sizes (e.g. Cohen's *d*, Pearson's *r*), indicating how they were calculated
	☒	Clearly defined error bars<br>*State explicitly what error bars represent (e.g. SD, SE, CI)*

Our web collection on statistics for biologists may be useful.

Software and code

Policy information about availability of computer code

Data collection	NMR experiments were carried out at 298K on Varian INOVA 600 and 900 MHz spectrometers at the UC Denver NMR Core facility. X-ray diffraction data were collected at the ALS 4.2.2 beamline, Berkeley administrated by the Molecular Biology Consortium. Cells were imaged live on a ZeissAxiovert 200M inverted microscope fitted with a 40x oil objective. Flow cytometry samples were collected on a FACScan instrument. Fluorescence polarization was conducted on a Tecan infinite M1000Pro. FRET data was collected on a Horiba Scientific Fluoromax 4.
Data analysis	NMRPipe Suite and other software listed in the Method section. NMRPipe, CcpNmr Suite v2.1. Software for structure determination include HKL2000, COOT v0.9, and PHENIX v1.18 as listed in Method section. Cell images were acquired with Slidebook (v6.0.18) and exported for analysis in ImageJ (v1.51). mCherry foci sizes were calculated using ImageJ image processing program (v1.51) using the Analyze Particle tool. Data was plotted with R (v3.4.1) using ggplot2 (v3.2.1). Cell size and mCherry foci count per cell were calculated using CellProfiler cell image analysis software (v3.1.5) to identify nuclei and mCherry foci, relate mCherry foci to nuclei containing them and measure the size of nuclei and number mCherry foci contained. Data was plotted with R (v3.4.1) using ggplot2 (v3.2.1). Flow cytometry samples was analyzed using FlowJo (v10). Fluorescence polarization was collected using Tecan i-control. FRET data was collected using FuorEssence v3.5 and processed using Matlab R2018a.

For manuscripts utilizing custom algorithms or software that are central to the research but not yet described in published literature, software must be made available to editors/reviewers upon request. We strongly encourage code deposition in a community repository (e.g. GitHub). See the Nature Research guidelines for submitting code & software for further information.

Data

Policy information about availability of data

All manuscripts must include a data availability statement. This statement should provide the following information, where applicable:

- Accession codes, unique identifiers, or web links for publicly available datasets
- A list of figures that have associated raw data
- A description of any restrictions on data availability

Coordinates and structure factors for the ATPaseCW cassette have been deposited in the Protein Data Bank (PDB ID 7K7T [https://www.rcsb.org/structure/7K7T]). All relevant data supporting the key findings of this study are available within the article and its Supplementary Information files or from the corresponding author upon reasonable request. Source data are provided with this paper. A reporting summary for this article is available as a Supplementary Information file.

Field-specific reporting

Please select the best fit for your research. If you are not sure, read the appropriate sections before making your selection.

☒ Life sciences ☐ Behavioural & social sciences ☐ Ecological, evolutionary & environmental sciences

For a reference copy of the document with all sections, see nature.com/authors/policies/ReportingSummary-flat.pdf

Life sciences study design

All studies must disclose on these points even when the disclosure is negative.

Sample size	present in relevant figure legends. The sample number was not predetermined using statistical analysis. The common practice in the field for measuring the number and size of nuclear bodies is to measure between 50-100 nuclei. We made sure to equal or exceed the upper bound of common practice.
Data exclusions	no data exclusions
Replication	present in relevant figure legends
Randomization	no randomization
Blinding	no blinding

Reporting for specific materials, systems and methods

Materials & experimental systems

n/a	Involved in the study	
☒	☐	Unique biological materials
☐	☒	Antibodies
☒	☐	Eukaryotic cell lines
☒	☐	Palaeontology
☒	☐	Animals and other organisms
☒	☐	Human research participants

Methods

n/a	Involved in the study	
☒	☐	ChIP-seq
☒	☐	Flow cytometry
☒	☐	MRI-based neuroimaging

Antibodies

Antibodies used	Antibodies used for assays: Anti-GST (13-0022, 1:1000) antibody was from EpiCypher and anti-Rabbit-HRP (NA934V, 1:20,000) antibody was from GE.
Validation	All antibodies validation are available on the manufacturers' websites.
