# Supplementary material for: Molecular mechanism of the MORC4 ATPase activation
Source: Nat Commun. 2020 Oct 29;11:5466. doi: 10.1038/s41467-020-19278-8 (PMC7596504; doi:10.1038/s41467-020-19278-8)
Supplement: Supplementary file 4 — Description of Additional Supplementary Files [file 41467_2020_19278_MOESM4_ESM.pdf]

Description of Additional Supplementary Files

Supplementary Data 1: List of averages and standard deviations for peptides in histone peptide microarrays.
