# Supplementary material for: Molecular mechanism of the MORC4 ATPase activation
Source: Nat Commun. 2020 Oct 29;11:5466. doi: 10.1038/s41467-020-19278-8 (PMC7596504; doi:10.1038/s41467-020-19278-8)
Supplement: Supplementary file 5 — Supplementary Data 1 [file 41467_2020_19278_MOESM5_ESM.pdf]

Average	SD	Peptide
0.336	0.070	H3 (1-20)
0.457	0.084	H3K14ac
0.417	0.072	H3K9ac
0.409	0.077	H3K4ac
0.320	0.091	H3K4ac + K14ac
0.299	0.093	H3K9ac + K14ac
0.315	0.085	H3K4ac + K9ac
0.198	0.084	H3K4ac + K9ac + K14ac
0.254	0.097	H3K18ac
0.150	0.077	H3K14ac + K18ac
0.277	0.097	H3K9ac + K18ac
0.284	0.082	H3K4ac + K18ac
0.356	0.110	H3K9ac + K14ac + K18ac
0.255	0.079	H3K4ac + K14ac + K18ac
0.383	0.096	H3K4ac + K9ac + K18ac
0.392	0.092	H3K4ac + K9ac + K14ac + K18ac
0.719	0.129	H3K4me3
0.907	0.096	H3K4me3 + K9ac
0.533	0.102	H3K4me3 + K14ac
0.584	0.073	H3K4me3 + K18ac
0.580	0.102	H3K4me3 + K9ac + K14ac
0.456	0.138	H3K4me3 + K9ac + K18ac
0.570	0.161	H3K4me3 + K14ac + K18ac
0.708	0.149	H3K4me3 + K9ac + K14ac + K18ac
0.326	0.073	H3T3p + K4me3 + K9ac + K14ac + K18ac
0.368	0.085	H3T3p + K4me3
0.273	0.083	H3R2me2a + T3p + K4me3 + K9ac + K14ac + K18ac
0.169	0.070	H3R2me2a + T3p + K4me3
0.687	0.105	H3R2me2a + K4me3
0.490	0.078	H3K4me2
0.441	0.110	H3K4me2 + K9ac + K14ac + K18ac
0.349	0.108	H3K4me1
0.264	0.063	H3K4me1 + K9ac + K14ac + K18ac
0.203	0.107	H3S10p
0.202	0.100	H3K4ac + K9ac + S10p + K14ac + K18ac
0.501	0.092	H3K4me3 + S10p
0.420	0.113	H3K4me3 + K9ac + S10p + K14ac + K18ac
0.595	0.117	H3R2me2a + K4me3 + S10p
0.663	0.080	H3R2me2a + K4me3 + K9ac + S10p + K14ac + K18ac
0.498	0.092	H3K9me3
0.459	0.107	H3K4ac + K9me3 + K14ac + K18ac
0.560	0.103	H3K4me2 + K9ac + K18ac
0.420	0.107	H3K4me1 + K9ac + K18ac
0.246	0.120	H3R2me2a
0.302	0.093	H3R2me2a + K4ac + K9ac + K14ac + K18ac
0.423	0.070	H3R2me2a + K4me3 + K9ac + K14ac + K18ac
0.483	0.159	H3R2me1 + K4me3

0.568	0.106	H3R2me1 + K4me3 + K9ac + K14ac + K18ac
0.413	0.110	H3Cit2
0.638	0.117	H3Cit2 + K4me3
0.789	0.171	H3Cit2 + K4me3 + K9ac + K14ac + K18ac
0.183	0.030	H3Cit2 + K4ac + K9ac + K14ac + K18ac
0.206	0.103	H3T3p
0.218	0.099	H4 (1-23)
0.117	0.092	H4K5ac + K8ac + K12ac + K16ac
0.321	0.114	H3R2me2a + K4me2
0.391	0.091	H3R2me2s + K4me2
0.527	0.089	H3R2me1 + K4me2
0.625	0.114	H3Cit2 + K4me2
0.302	0.061	H3K4N3
0.466	0.081	H4K5ac
0.379	0.111	H4K8ac
0.397	0.059	H4K12ac
0.336	0.107	H4K16ac
0.354	0.111	H4K5ac + K12ac
0.306	0.103	H4K8ac + K16ac
0.281	0.088	H4K5ac + K8ac + K12ac
0.269	0.102	H4R3me2a
0.160	0.105	H4R3me2s
0.222	0.098	H4R3me1
0.114	0.086	H4S1p + R3me2a
0.064	0.071	H4S1p + R3me2s
0.262	0.087	H4S1p + R3me1
0.247	0.117	H4R3me2a + K5ac + K8ac + K12ac + K16ac + K20ac
0.323	0.115	H4R3me2s + K5ac + K8ac + K12ac + K16ac + K20ac
0.427	0.106	H4R3me1 + K5ac + K8ac + K12ac + K16ac + K20ac
0.382	0.098	H4K20me3
0.124	0.103	H4K20me2
0.098	0.097	H4K20me1
0.251	0.103	H4K12ac + K16ac + K20me3
0.277	0.085	H4K12ac + K16ac + K20me2
0.224	0.101	H4K12ac + K16ac + K20me1
0.352	0.089	H4K12ac + K16ac
0.752	0.100	H3K4me3 + R8me2s + K9me3
0.189	0.119	H3 (15-41)
0.384	0.081	H3K18me3
0.438	0.124	H3K36me3
0.390	0.087	H3K18me3 + K36me3
0.811	0.100	H3K4me3 + R8me2a + K9me3
0.381	0.107	H4 (11-27)
0.358	0.101	H3 (74-84) N-ac
0.427	0.086	H3K79me3
0.279	0.107	H3K79me2
0.197	0.109	H3K79me1
0.257	0.102	H3 (74-84)

0.098	0.094	H3K36me3 (27-45)
0.273	0.093	H3K36me2 (27-45)
0.213	0.105	H3K36ac (27-45)
0.229	0.099	H3 (27-45)
0.282	0.110	H3T3p
0.186	0.078	H3.3K36me1
0.226	0.096	H3K9me2 + K27me2
0.554	0.118	H3K4me3 + K9me3
0.407	0.119	H3K9me2
0.335	0.098	H3K9me1
0.328	0.076	K3K4me1 + K18ac
0.321	0.086	H3T11p
0.243	0.099	H3K18me3
0.364	0.105	H3K18me2
0.300	0.080	H3K18me1
0.354	0.109	H3R8me1
0.393	0.093	H3R8me2a
0.203	0.136	H3R8me2s
0.254	0.075	H3K9ac + S10p
0.176	0.080	H3K9me3 + S10p
0.220	0.080	H3K9me2 + S10p
0.290	0.108	H3K9me1 + S10p
0.467	0.037	H3K4me3 + K9ac + S10p
0.458	0.077	H3R17me2a
0.454	0.107	H3R17me2s
0.432	0.070	H3R17me1
0.246	0.086	H3K14me2
0.292	0.109	H3K14me3
0.808	0.106	H3R2me2s + K4me3
0.375	0.103	H3K18ac (1-25)
0.340	0.139	H3R17me2a (1-25)
0.318	0.109	H3R17me2a + K18ac (1-25)
0.459	0.118	H3 (1-25)
0.238	0.119	H3T6p
0.247	0.097	H3K4me3 + T6p
0.214	0.107	H3K4me2 + T6p
0.096	0.079	H3T6p + K9ac + K14ac + K18ac
0.198	0.094	H3K4me3 + T6p + K9ac + K14ac + K18ac
0.234	0.082	H3K4me2 + T6p + K9ac + K14ac + K18ac
0.353	0.098	H3K18ac + K23ac + K27ac
0.201	0.037	H3K18ac + K23ac + K27ac + S28p
0.254	0.095	H3K23ac
0.678	0.092	H3R2me2s + K4me3 + K9ac + K14ac + K18ac
0.433	0.085	H3R8me1 + K9me3
0.497	0.109	H3R8me1 + K9me2
0.350	0.094	H3R8me2a + K9me3
0.232	0.115	H3R8me2a + K9me2
0.233	0.088	H3R8me2a + K9me1

0.283	0.109	H3R8me2s + K9me3
0.360	0.107	H3R8me2s + K9me2
0.275	0.086	H3R8me2s + K9me1
0.263	0.100	H3K4ac + K9me2 + K14ac + K18ac
0.278	0.101	H3K4ac + K9me1 + K14ac + K18ac
0.312	0.107	H3K27me3
0.189	0.086	H3K27me2
0.279	0.103	H3K27me1
0.230	0.108	H3R26me2a + K27me3
0.179	0.100	H3R26me2a + K27me1
0.306	0.105	H3 15-34
0.348	0.143	H3 30-49
0.183	0.089	H3 105-124
0.225	0.052	H3.3 15-34
0.279	0.098	H3.3 30-49
0.338	0.064	H3.3 75-94
0.406	0.118	H3 120-135
0.161	0.057	H3 15-34
0.241	0.120	H3K9-Nle (1-20)
0.372	0.092	H3K14-Nle (1-20)
0.232	0.115	H3K18-Nle (11-26)
0.223	0.103	H3K23-Nle (15-31)
0.233	0.099	H3T6p + K9me3
0.244	0.104	H3T6p + R8me2a + K9me3
0.217	0.092	H3R26me2a
0.413	0.109	H3K27me3 + S28p
0.346	0.096	H3K27me2 + S28p
0.317	0.118	H3K4ac + K9me3
0.255	0.124	H3K9me2 (1-32)
0.344	0.120	H3K9me2 + K27me1 (1-32)
0.269	0.083	H3K27ac
0.317	0.077	H3K27ac + S28p
0.396	0.116	H3S28p
0.322	0.112	H3 (1-20) N-ac
0.355	0.102	H3 (52-61)
0.249	0.057	H3K56ac (52-61)
0.189	0.052	H3K56me3 (52-61)
0.223	0.108	H3K9me2 (1-15)
0.536	0.118	H3K4me2 + K9me2
0.554	0.113	H3K4me1 + K9me2
0.550	0.094	H3K4me3 + K9me2
0.155	0.116	H3K4A + K9me2 (1-15)
0.240	0.091	H2A (1-17)
0.235	0.087	H2AK5ac + K9ac + K13ac + K15ac
0.236	0.112	H2AK5ac
0.368	0.092	H2AS1p + K5ac
0.360	0.114	H2AR3me2a + K5ac
0.273	0.103	H2AS1p + R3me2a + K5ac

0.168	0.123	H2ACit3 + K5ac
0.144	0.081	H2AS1p + Cit3 + K5ac
0.208	0.093	H2AS1p + K5ac + K9ac + K13ac + K15ac
0.303	0.069	H2AK5ac + K9ac + K13ac + K15ac (no N-ac)
0.240	0.109	H2AS1p + K5ac + K9ac + K13ac + K15ac (no N-ac)
0.179	0.111	H2AX (132-142)
0.208	0.119	H2AX (S139p)
0.278	0.113	H2A.Z (1-19)
0.264	0.123	H2A.Z (1-19) N-ac
0.206	0.095	H2A.Z K4ac + K8ac + K12ac (1-19)
0.269	0.078	H2A.Z K4ac + K8ac + K12ac (1-19) N-ac
0.316	0.118	H3Cit8 (1-21)
0.388	0.116	H3 (21-44)
0.255	0.080	H3K36me1 (21-44)
0.334	0.093	H3K36me2 (21-44)
0.341	0.077	H3K36me3 (21-44)
0.301	0.140	H3K36ac (21-44)
0.216	0.118	H3K27acK36me1 (21-44)
0.367	0.083	H3K27acK36me2 (21-44)
0.170	0.095	H3K27acK36me3 (21-44)
0.172	0.059	H2BK120ub1 (Leu-Arg-Gly-Gly) (108-125)
0.174	0.120	H2B (108-125)
0.294	0.081	H4 (1-25)
0.357	0.080	H4K20me3 (1-23)
0.284	0.082	H4K12acK16acK20me3 (1-25)
0.208	0.083	H4R3me2a + K5ac
0.353	0.061	H4 (1-23) (no N-ac)
0.420	0.039	H4K20ac
0.201	0.076	H4S1p + K5ac + K8ac + K12ac + K16ac
0.320	0.088	H4S1p (1-23)
0.254	0.118	H4K5ac + K8ac
0.253	0.080	H4K5ac + K16ac
0.332	0.068	H4K5ac + K20ac
0.189	0.105	H4K8ac + K12ac
0.356	0.059	H4K8ac + K20ac
0.219	0.063	H4K12ac + K16ac
0.213	0.109	H4K12ac + K20ac
0.390	0.051	H4K16ac + K20ac
0.334	0.108	H4K12ac + KQ5,8,16,20
0.231	0.115	H4K5me1
0.283	0.118	H4K8me1
0.347	0.110	H4K12me1
0.386	0.071	H4K5ac + K8me1 + K12ac
0.337	0.113	H4K5me1 + K8ac + K12me1
0.359	0.091	H4K5me1 + K8me1 + K12me1
0.329	0.111	H4K8ac + K12ac + K16ac
0.320	0.116	H4K5ac + K12ac + K16ac
0.239	0.120	H4K5ac + K8ac + K16ac

0.161	0.105	H4K5me1 + K8ac + K12ac + K16ac
0.245	0.097	H4K5ac + K8me1 + K12ac + K16ac
0.263	0.102	H4K5ac + K8ac + K12me1 + K16ac
0.387	0.094	H2B (1-24)
0.428	0.118	H2BK5me3
0.349	0.079	H2BK5me2
0.219	0.089	H2BK5me1
0.238	0.099	H2BK12ac
0.365	0.097	H2BK15ac
0.277	0.096	H2BK16ac
0.397	0.117	H2BK20ac
0.224	0.057	H2BK12ac + K15ac + K16ac + K20ac
0.168	0.077	yH2A (10-25)
0.184	0.118	H2A (10-25)
0.207	0.098	H2A.X (1-17)
0.246	0.118	H2A.X K5ac
0.694	0.188	H3K4me3 (1-11)
0.407	0.089	H3K4me3 (1-7)
0.275	0.095	H3K36me3
0.392	0.095	H3S31p + K36me3
0.273	0.117	H3K23me3 (15-34)
0.080	0.066	H3K23me2 (15-34)
