# Supplementary material for: Molecular mechanism of the MORC4 ATPase activation
Source: Nat Commun. 2020 Oct 29;11:5466. doi: 10.1038/s41467-020-19278-8 (PMC7596504; doi:10.1038/s41467-020-19278-8)
Supplement: Supplementary file 2 — Peer Review File [file 41467_2020_19278_MOESM2_ESM.pdf]

Reviewers' comments:

Reviewer #1 (Remarks to the Author):

This manuscript presents an important advance in understanding the molecular details of how MORC4 ATPase regulates chromatin, and how it compares to the related MORC2 and MORC3 ATPases. This family of proteins plays critical roles in normal and pathological chromatin biology and an understanding of their specific roles will enhance our understanding of related biological processes. The data presented here represent an excellent basis from which to further probe these proteins including structural data/insight and specific mutations that modulate precise biochemical activities. I support publication of this work after the following points are addressed.

1. Label Fig 1a with the construct boundaries and names used in the paper (CW, ATPaseCW, etc)

2. Page 4, line 88-89 "Neither methylated histone peptide H3K4me3 nor unmodified H3 peptide (both aa 1-12 of H3) stimulated a further increase in the rate of ATP hydrolysis, implying that the enzymatic activity is largely histone independent." This last conclusion cannot be made because only two histone peptides/states have been tested. Neither is it clear at this point in the paper why only H3K4me3 was chosen to test. I'd suggest that the histone binding aspects be moved to the section on page six, where the full set of histone peptides is tested and the rationale for H3K4me3 is provided.

3. Figure 2f,g figure caption and lines 51-52 of page 7 should specific tryptophan fluorescence, (as opposed to simply fluorescence).

4. The label on figure 3b seems to be in consistent with text and figure legend. The label indicates that the CW:ATPase ratio is constant and the peptide is being titrated, whereas the text/figure legend indicates the ATPase is being titrated in.

5. The overall quality metrics for the X-ray structure are somewhat lower than expected for a 2.9A resolution structure. Have the authors been able to improve the refinement and Rfree/clash scores shown in the preliminary PDB report and Supp table 1 ?

Reviewer #2 (Remarks to the Author):

The manuscript by Tencer et al. reports a study on the structure and function of the ATPase-CW cassette domain of MORC4, a GHKL ATPase protein that is linked to diverse pathologies. The study identifies a striking difference in DNA binding ability and ATPase activation with respect to the homologous MORC3 domain, and reports interesting observations on the impact on nucleosome stability and cell cycle progression. The new insights offered are however incremental and seem best suited for a more specialist journal.

This study follows up on the lab's earlier work on the MORC3 ATPaseCW domain (ref. 15) that presented a model for MORC3 activation based on structural and biochemical studies. The current study demonstrates that the MORC4 CW domain has a positive exposed patch that can bind DNA, and that this is required for activation. Yet, as a whole, the mechanism of activation remains largely unclear and requires more structural and mutagenesis data to understand whether DNA binding to the ATPase domain involves the same or another surface as in MORC3, how the CW and ATPase DNA binding activities cooperate within the ATPaseCW construct, whether/what conformational change is induced upon activation, and what role H3 tail binding plays particularly since it cannot be seen in the crystal structure despite its high binding affinity.

The functional data presented are based on MORC4 over-expression and should be treated with

caution. The conclusion that MORC4 plays a negative role in S-phase progression (p.13) is unwarranted at this point. The rationale for investigating cell cycle progression based on reduced DNA unwrapping is not convincing.

The manuscript does not provide sufficient context for the wider audience to understand the relevance and rationale behind some of the findings and experiments, e.g. the meaning of the conformational change or the dimeric nature of the crystal structure. Reading of ref. 14 and 15 is required to properly interpret the findings. To make things worse, references to the lab's earlier work on the MORC3 ATPaseCW domain (ref. 15) are missing throughout the paper. The conflicting data with respect to ref. 14 on the intrinsic ATPase activity of MORC3 also remain an open question.

The authors should take more care in their phrasing to prevent over-stating their findings. For example, the conclusion that MORC3 ATPaseCW domain stabilized the unwrapped state of the nucleosome (p. 10) is not supported by the data given the error-bars in the S1/2 measurements. Also, on p. 9 the enhancement of H3 tail binding affinity in presence of DNA is interpreted as a sign that the DNA "…. releases at least in part the H3K4me3-binding site of CW", while this could also be an electrostatic effect, especially considering the high affinity in absence of DNA does not provide a clear indication that binding site is occluded.

The EMSA assays are below publication quality.

Reviewer #3 (Remarks to the Author):

The authors present a biochemical and structural characterization of MORC4, a member of the MORC family, which almost remains uncharacterized, even though it is associated with several diseases. The authors focused their research on elucidating the molecular mechanism for MORC4 activation. By using enzymatic and binding assays, the authors show for the first time that the MORC4 presents a) DNA dependent ATPase activity, b) ability to discriminate PTM on histone tails, c) presents a unique DNA binding capability and d) presents enhanced nucleosome stabilization to transcription factors. Also, MORC4 presents an in vivo function e) on phase separation and f) cell cycle progression function by in-cell experiments. The report presents novels and interesting functional data of MORC4. However, the work lacks certain controls and the characterization of substrates as well as mutants that would clarify the mechanism of action of MORC4. I am positive to consider the paper for publication if authors provide the controls, experiments, and corrections suggested. Here are my main comments

Major:

The nucleosome substrate presented in Figure 5C corresponds to the LexA, not to the Gal4 (it should be at least 30 bases between Cy3 and the Gal binding site). Also, on the Gal4 substrate, the distance between the Cy3-Cy5 is almost 90 Å (based on crude modeling, and assuming a B DNA conformation of the 30bp linker). Keeping this distance in mind, can a productive FRET signal be observed? See the attached figure (75 bp linker is not present in the model).

Since MORC4 presents a high affinity for free double-strand DNA, why the nucleosome substrates have long DNA overhangs? The 75bp 3' linker is used in the cited paper to attach nucleosomes to the sensor surface. However, the assays are performed in solution. I recommend repeating the experiments with nucleosomes that contain no/minimal DNA overhangs. That may differentiate DNA binding versus chromatin binding.

What is the minimum dsDNA length that can activate the ATPase activity of MORC4 ATPaseCW? Also, what is the minimum dsDNA length that can support MORC4ATPase DNA binding? The fully extended 601 DNA is significantly longer than the distance between the ATPase and CW domains. Therefore,

multiple MORC4 can bind to this extensive DNA sequence. Can MORC4 ATPaseCW bind to the overhangs used in on the presented experiment?

Does Morc4 binding to nucleosome activate the ATPase activity? Also, since the affinity H3 peptide increase with K4 methylation. What is the affinity of MORC4 for H3K4me(1-3) nucleosomes?

The curve for MORC3 presented in Figure 5F shows significant variability in the measurement of the dFRET signal, and it contains 30 times more protein than MORC4, making the experiment challenging to interpret. Plus, the active substrate for MORC3 should be H3K4me3 nucleosomes. This data should be removed.

The FRET experiment in Figure 5E was performed with 0.3 mM ATP. However, the assay presented in Figure 3F was performed without ATP. It seems that the ATPase activity of MORC4 is not critical for the stabilization function, but without the appropriate controls, it is difficult to establish the mechanistic effect ATPase activity versus just the DNA or chromatin binding. Please include control experiments (With and without ATP).

For the in-vivo experiments (Figure 6), the authors used a different set of mutants than in the biochemistry section. The authors should include the CWkrr mutant in the analysis. Also, there no experimental evidence on the E56A ether disrupts ATPase activity or/and retains DNA binding. This data should be presented.

In Figure 6C, the authors observe a small increase in the percentage of cells in S phase when over expressed MORC4. The mutants used decreased the rate of cells in this phase, but the levels are not comparable to the mock control. So, what is the effect of the MORC4 double mutant on the cell cycle? Also, what is the impact of the MORC4 CWkrr on cell cycle? Do the authors know the distribution of MORC4 during the cell cycle?

In line 49, the authors quote that over expression of MORC4 in breast cancer tissue is linked with poor patient survival. Contrary, microRNAs targeting MORC4 decreased cell growth and activation of apoptosis (ref. 9). How the previous statement is compatible with what is presented in line 316, in which MORC4 plays a negative role in S-phase progression. The expected effect on tumor growth should be the opposite, particularly were cancer cells require more transcriptional activation for cell growth. The authors should clarify it with the proposed mechanism.

Minor:

In Figure 1A, the authors should include the sequence registry and length of the constructs used in the study for clarity.

On cartoon representation of the MORC4 X-ray structure (Figure 1D), it is evident that there are several unstructured loops. Those regions should be represented in dashed lines. Also, residues mutated in this study should be shown in the context of the structure.

Since the MORC family present significant identity (about 55 % between MORC4 and MORC3), the authors should show sequence alignment of the family, highlighting the sequence differences.

The authors should comment on the unsuccessful attempt to co-crystalize the MORC4 with H3K4me3 peptide, despite the promising peptide binding experiments. Also, it is not clear what is the effect of PTM peptides (particularly H3K4me) on MORC4 activity.

Regarding MORC4 binding to histones, it seems that just the methylation on H3K4 is the only significant event rather than the methylation state (me1, me2, or me3) (Figures 2A and 2C). Can be

the potential peptide-binding site on the CW identified? There is a very distinct binding site configuration for me1, me2, or me3.

Which program or algorithm has been used to generate these panels on Figures 3F and 3G? APBS is recommended for this task. Since the CW structure misses some loop regions, have completed those for the figure purpose?

The authors should calculate the apparent Kds for the binding isotherms (gel shifts) presented in Figures 4E, 4F, 4G, and 4H. It will help to compare with other biochemistry experiments. Also, there is no description of what it is the meaning of the colored rectangle? 50% of the change in the signal of 601 DNA)?

The authors should label the rows of the image (DPI, mCheery?) in Figure 6.

In supplementary Figure 3, the complex seems to disappear at higher MORC4 ATPaseCWkrr. Does the protein/DNA complex remain on the wells or there is degradation?

Can apparent Kd been calculated for the experiment presented Supplementary Figure 4, to be compared with the DNA biding Kd presented Figure 5?

We thank the Editor and Reviewers for the insightful and very constructive comments, which were helpful in revising and strengthening this manuscript.

In the revised manuscript, new data are shown in Figs. 1d, 5a, 5b, 5c, 5d, 5e, 6d, 6e, 7c and Suppl. Figs. 1, 2, 6, 7, 10, 12 and 13. In addition, Figs. 1a, 3b, 3f, 3g, 4e-h, 6c and 6f have been revised.

Reviewer 1, Comment 1: Label Fig 1a with the construct boundaries and names used in the paper (CW, ATPaseCW, etc).

> **Author's response:** we have added labels and also expanded Fig.1a legend.

Reviewer 1, Comment 2: Page 4, line 88-89 "Neither methylated histone peptide H3K4me3 nor unmodified H3 peptide (both aa 1-12 of H3) stimulated a further increase in the rate of ATP hydrolysis, implying that the enzymatic activity is largely histone independent." This last conclusion cannot be made because only two histone peptides/states have been tested. Neither is it clear at this point in the paper why only H3K4me3 was chosen to test. I'd suggest that the histone binding aspects be moved to the section on page six, where the full set of histone peptides is tested and the rationale for H3K4me3 is provided.

> **Author's response:** to keep comparison of the ATPase activity in one place, we have added an explanation after this sentence: "…(we discuss binding of CW to H3 below)."

Reviewer 1, Comment 3: Figure 2f,g figure caption and lines 51-52 of page 7 should specific tryptophan fluorescence, (as opposed to simply fluorescence). – done

The label on figure 3b seems to be in consistent with text and figure legend. The label indicates that the CW:ATPase ratio is constant and the peptide is being titrated, whereas the text/figure legend indicates the ATPase is being titrated in. – the label has been corrected, thank you.

The overall quality metrics for the X-ray structure are somewhat lower than expected for a 2.9A resolution structure. Have the authors been able to improve the refinement… – as suggested, the structure refinement and clash scores have been improved.

Reviewer 2, Comment 1: the mechanism of activation remains largely unclear and requires more structural and mutagenesis data to understand whether DNA binding to the ATPase domain involves the same or another surface as in MORC3, how the CW and ATPase DNA binding activities cooperate within the ATPaseCW construct, whether/what conformational change is induced upon activation, and what role H3 tail binding plays particularly since it cannot be seen in the crystal structure despite its high binding affinity.

> **Author's response:** to clarify the mechanism of action we have performed additional experiments
>
> -mapping the DNA binding interface: in addition to mutating K460/R462/R463 in the CW domain, we have mutated nine residues in the ATPase domain. We have generated K213Q/K214Q, R225Q/K227Q/K314Q, K352Q/R355Q, and K401Q/K403Q mutants of the ATPaseCW cassette and tested binding of these mutants to DNA by EMSA (the data are shown in new Fig. 5 and described on pages 9-10).
>
> -we have compared binding of the ATPaseCW cassette and the ATPase domain to NCP_{207} and NCP_{147}. We found that both bind better to the nucleosome containing longer linker DNAs. The

new data are shown in Suppl. Fig. 7, and the text on page 10 has been added/revised.

-we found that the presence of ATP is essential for MORC4 to stabilize the nucleosome in FRET assay (new FRET data are shown in Fig. 6d, e).

-EMSA assays (Fig. 4e-h) suggest that binding to H3 peptide enhances binding of the ATPaseCW cassette to 601 DNA.

-clarified that ‘the same surface of CW is involved in binding to the ATPase domain and H3K4me3[13-15] (page 5). Therefore, it's not surprising that electron density for the peptide is not present in the ATPase:CW complex- CW binds to either the ATPase domain or histone tail, but not both simultaneously.

-we have removed reference to a conformational change.

Reviewer 2, Comment 2: The functional data presented are based on MORC4 over-expression and should be treated with caution. The conclusion that MORC4 plays a negative role in S-phase progression (p.13) is unwarranted at this point. The rationale for investigating cell cycle progression based on reduced DNA unwrapping is not convincing.

Author’s response: we have repeated the cell cycle experiments and obtained very reproducible results (new data are shown in Fig. 7c and Suppl. Figs. 12 and 13). We have also added the dual and triple mutants, toned down the conclusion, and revised the rationale.

Reviewer 2, Comment 3: The manuscript does not provide sufficient context for the wider audience to understand the relevance and rationale behind some of the findings and experiments, e.g. the meaning of the conformational change or the dimeric nature of the crystal structure.

Author’s response: we have removed reference to a conformational change. The dimeric crystal structures of the ATPase-CW regions of MORC3 and MORC2 and the dimeric interfaces have been investigated in previous studies. The following sentence has been revised on page 5: “…a large dimer interface, which was also observed in the respective structures of MORC3 and MORC2[14,15,17].”

… references to the lab’s earlier work on the MORC3 ATPaseCW domain (ref. 15) are missing throughout the paper. - we cite ref. 15 on pages 3, 5, 6 and 13.

Reviewer 2, Comment 4: … the conclusion that MORC3 ATPaseCW domain stabilized the unwrapped state of the nucleosome (p. 10) is not supported by the data given the error-bars in the S1/2 measurements. – these data have been removed as suggested by Reviewer 3.

Also, on p. 9 the enhancement of H3 tail binding affinity in presence of DNA is interpreted as a sign that the DNA “…. releases at least in part the H3K4me3-binding site of CW”, while this could also be an electrostatic effect... – the phrase “… releases at least in part…” has been removed.

Reviewer 3, Comment 1: The nucleosome substrate presented in Figure 5C corresponds to the LexA, not to the Gal4 (it should be at least 30 bases between Cy3 and the Gal binding site). Also, on the Gal4 substrate, the distance between the Cy3-Cy5 is almost 90 Å (based on crude modeling, and assuming a B DNA conformation of the 30bp linker). Keeping this distance in mind, can a productive FRET signal be observed? See the attached figure (75 bp linker is not present in the model).

Author's response: Fig. 6c has been revised to show the Gal4-NCP model (it was LexA-NCP_{147}).

Reviewer 3, Comment 2: Since MORC4 presents a high affinity for free double-strand DNA, why the nucleosome substrates have long DNA overhangs? The 75bp 3' linker is used in the cited paper to attach nucleosomes to the sensor surface. However, the assays are performed in solution. I recommend repeating the experiments with nucleosomes that contain no/minimal DNA overhangs.

Author's response: as suggested, we have examined binding of the ATPase and CW domains and the ATPaseCW cassette to NCP_{147}. Binding of ATPaseCW was decreased 2-fold, whereas binding of the ATPase domain was decreased 5-fold, compared to binding of these constructs to NCP_{207}. These results suggest that extra-nucleosomal DNA enhances binding of MORC4. The new data are shown in Suppl. Fig. 7 and the text on page 10 has been added/revised.

Reviewer 3, Comment 3: What is the minimum dsDNA length that can activate the ATPase activity of MORC4 ATPaseCW? Also, what is the minimum dsDNA length that can support MORC4ATPase DNA binding? The fully extended 601 DNA is significantly longer than the distance between the ATPase and CW domains. Therefore, multiple MORC4 can bind to this extensive DNA sequence. Can MORC4 ATPaseCW bind to the overhangs used in on the presented experiment?

Author's response: we have carried out EMSA experiments to determine the minimum dsDNA length- it's ~30 bp. The data are shown in new Suppl. Fig. 6 and described on page 10.

Reviewer 3, Comment 4: Does Morc4 binding to nucleosome activate the ATPase activity? Also, since the affinity H3 peptide increase with K4 methylation. What is the affinity of MORC4 for H3K4me(1-3) nucleosomes?

Author's response: we have performed ATPase assays with the nucleosome (new Suppl. Fig. 1), the data demonstrate that the nucleosome stimulates the ATPase activity. We were unable to perform binding experiments with mono/di/tri-methylated NCPs because these nucleosomes are prohibitively expensive to us.

Reviewer 3, Comment 5: The curve for MORC3 presented in Figure 5F shows significant variability in the measurement of the dFRET signal… This data should be removed.

Author's response: as suggested, these data have been removed.

Reviewer 3, Comment 6: The FRET experiment in Figure 5E was performed with 0.3 mM ATP. However, the assay presented in Figure 3F was performed without ATP. It seems that the ATPase activity of MORC4 is not critical for the stabilization function, but without the appropriate controls, it is difficult to establish the mechanistic effect ATPase activity versus just the DNA or chromatin binding. Please include control experiments (With and without ATP).

Author's response: we have performed FRET assays with and without ATP (new data are shown in Fig. 6e) and found that the presence of ATP is essential (described on page 11).

Reviewer 3, Comment 7: For the in-vivo experiments (Figure 6), the authors used a different set of mutants than in the biochemistry section. The authors should include the CWkrr mutant in the analysis. Also, there no experimental evidence on the E56A ether disrupts ATPase activity or/and retains DNA binding. This data should be presented.

In Figure 6C, the authors observe a small increase in the percentage of cells in S phase when over expressed MORC4. The mutants used decreased the rate of cells in this phase, but the levels are not comparable to the mock control. So, what is the effect of the MORC4 double mutant on the cell cycle? Also, what is the impact of the MORC4 CWkrr on cell cycle? Do the authors know the distribution of MORC4 during the cell cycle?

> **Author's response:** we have tested the E56A mutant in ATPase activity assay and EMSA – it is catalytically inactive but retains the DNA binding activity (the new data are shown in Fig. 5f and Suppl. Fig. 10).
>
> We have repeated the cell cycle experiments and obtained very reproducible results (new data are shown in Fig. 7c and Suppl. Figs. 12 and 13). As suggested, we have included the dual and triple mutants. Due to a lack of verified commercially available antibodies we were unable to determine the cell cycle distribution of endogenous MORC4.

Reviewer 3, Comment 8: In line 49, the authors quote that over expression of MORC4 in breast cancer tissue is linked with poor patient survival. Contrary, microRNAs targeting MORC4 decreased cell growth and activation of apoptosis (ref. 9). How the previous statement is compatible with what is presented in line 316, in which MORC4 plays a negative role in S-phase progression. The expected effect on tumor growth should be the opposite, particularly were cancer cells require more transcriptional activation for cell growth. The authors should clarify it with the proposed mechanism.

> **Author's response:** MIR193-3p targets many other proteins known to regulate cell cycle including MYCN, KRAS, E2F6, DCAF7, HOXD13. Previous work has demonstrated a role for this miRNA as a tumor suppressor, and in breast cancer it is known to regulate KRAS expression (Grossi, Int J Genomics, 2017 PMID: 29038785). The previous work did not test MORC4 regulation of cell cycle or cell growth and using the miRNAs (and not just MORC4 expression) to do so has many compounding effects that would make interpreting those experiments difficult.

Reviewer 3, Comment 9:
Minor:
In Figure 1A, the authors should include the sequence registry and length of the constructs used in the study for clarity. - done

On cartoon representation of the MORC4 X-ray structure (Figure 1D), it is evident that there are several unstructured loops. Those regions should be represented in dashed lines. Also, residues mutated in this study should be shown in the context of the structure. – dashed lines are now included in Fig. 1d, and mutated residues are highlighted in Fig. 5a.

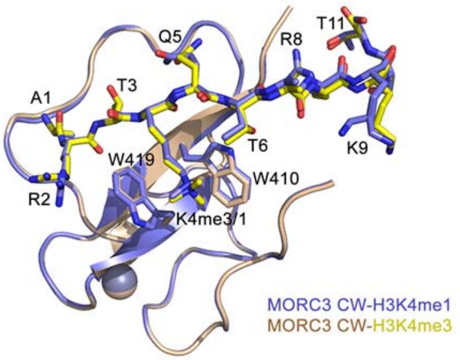

Since the MORC family present significant identity (about 55 % between MORC4 and MORC3), the authors should show sequence alignment of the family, highlighting the sequence differences. – the alignment is shown in Suppl. Fig. 2.

The authors should comment on the unsuccessful attempt to co-crystalize the MORC4 with H3K4me3 peptide, despite the promising peptide binding experiments. Also, it is not clear what is the effect of PTM peptides (particularly H3K4me) on MORC4 activity. – one possibility we were contemplating is that DNA needs to be present (although we did not obtain diffracting crystals in the presence of both DNA and the H3K4me3 peptide). To address this

issue, we are collaborating with the Cramer lab on determining the structure of the ATPaseCW cassette in complex with NCP by cryo-EM.

Regarding MORC4 binding to histones, it seems that just the methylation on H3K4 is the only significant event rather than the methylation state (me1, me2, or me3) (Figures 2A and 2C). Can be the potential peptide-binding site on the CW identified? There is a very distinct binding site configuration for me1, me2, or me3. – we have previously determined the crystal structures of the isolated CW domain of MORC3 in complex with the H3K4me3, H3K4me1 and H3unmod peptides (ref. 13). These peptides were bound in the same binding site, and K4 was positioned between two tryptophan residues in all complexes (please see figure above). The histone-binding site residues of MORC3 and MORC4 are highly conserved.

Which program or algorithm has been used to generate these panels on Figures 3F and 3G? APBS is recommended for this task. Since the CW structure misses some loop regions, have completed those for the figure purpose? – the electrostatic surface potential images were generated using APBS in Pymol with a range of –100/100 kT/e. The missing loops were completed. We have added this info in Fig. 3f, g legend.

The authors should calculate the apparent Kds for the binding isotherms (gel shifts) presented in Figures 4E, 4F, 4G, and 4H. It will help to compare with other biochemistry experiments. Also, there is no description of what it is the meaning of the colored rectangle? 50% of the change in the signal of 601 DNA)? …Can apparent Kd been calculated for the experiment presented Supplementary Figure 4, to be compared with the DNA biding K_d presented Figure 5? – we did attempt to calculate apparent K_ds but to avoid adding imprecise values decided not to use them.

The authors should label the rows of the image (DPI, mCheery?) in Figure 6. – done

In supplementary Figure 3, the complex seems to disappear at higher MORC4 ATPaseCWkrr. Does the protein/DNA complex remain on the wells or there is degradation? – it remains in the well.

Reviewer #1 (Remarks to the Author):

This manuscript is greatly improved. It is acceptable for publication now.

Reviewer #2 (Remarks to the Author):

The authors have improved the paper by including explicit references to previous work on MORC proteins which helps greatly to understand the rationale better. Still, the similarities between MORC4 and MORC3 could be and I think should be emphasized more to explain the activation mode to a wider audience. In particular, the fact that the MORC3 and MORC4 H3K4me binding site can be assumed to be similar based on sequence conservation helps to directly pinpoint the overlap of histone and ATPase binding sites in CW, which could be added to line 166. I think the crux of this to understanding the mode of activation is to find out why the H3 tail is capable of binding the ATPaseCW cassette in MORC3 but not in MORC4 and how that is related to the DNA binding of the CW in MORC4? An answer to that would fit very well in this journal. Another aspect where the similarity to MORC3 needs emphasis is the relevance of the dimerization, which is mentioned several times in this manuscript, i.e. in line 325/326 it is mentioned to be essential for activity of MORC4 without any proof, where in the case of MORC3 more data is available.

The new data on the DNA binding site within the ATPase domain are unclear. It is concluded that fig. 5b and 5c show most reduction in DNA binding, while, without any quantitation of the free DNA band intensity available, the mutants in Fig 5d and 5e seem to have the strongest reduction. Again it will be helpful to contrast with MORC3, should that data be available.

The FRET experiments lead to two conflicting conclusions: line 257/258 "This suggests that binding of the ATPaseCW cassette itself does not shift the equilibrium." but in line 265/266: "Collectively, these data reveal that binding of the MORC4 ATPaseCW cassette to the nucleosome shifts the unwrapping-wrapping equilibrium toward the wrapped state". This needs to be explained. Given the effect of ATP in the Gal4 assay, it should be investigated whether a similar effect hold for MORC4 binding itself.

The EMSA assays in fig 4 were already in the original manuscript.

The clarification on MORC3 H3 tail and ATPase binding site in is indeed helpful. As mentioned above it would best to directly relate this to MORC4. I don't see at all why it is not surprising that the H3 tail peptide can compete off the ATPase domain in MORC3 but not in MORC4. I would rather emphasize its absence as it highlights the functional difference between MORC3 and 4 and invest in figuring out the molecular reason for this, i.e. using a chimeric construct.

Other issues:
- line 104-111: a supplemental figure would be highly helpful here
- line 174: "band corresponding to the complex of CW with 601 DNA" is better rephrased to " a band smear corresponding to complexes of CW with 601 DNA" as multiple CW will associate to the long DNA substrate explaining the smeared appearance.
- line 176: "MORC3 was incapable of binding to 601 DNA" is better toned down since there is clear reduction of the free DNA bind indicating binding, even though perhaps the complexes precipitated or got stuck in the well which is not clearly visible in the picture
- line 203: "(both ATPase and CW domains are involved in this interaction)" a reference is needed here

Reviewer #3 (Remarks to the Author):

Thanks to the authors to take the time to answer the questions and requests for the reviews. I think the paper significantly improved in clarity and consistency. Also, the changes in the title and the in vivo result, helped to not overstate the results particularly by over expressing a protein not knowing the physiological levels. However, there are some minor comments that still need to be addressed I will support the publication of this manuscript, when the small comments are addressed.

In page 4-5, when the structure is described, but there is no figure for that. I recommend to add secondary structure to the top of alignment in Fig. Supple.2, including the secondary structure names (ej. α1, β8, etc).

In the section 'Extensive DNA binding site of MORC4 ATPaseCW Cassette", the authors identify two positive clusters in the structure surface, and speculate that since there are 40 Å away the surface is large. Authors should calculate the area and compare it with other DNA binding proteins.

The experiment of MORC4 ATPaseCW binding to Nucleosomes (Data not shown), Does the experiment was performed with the addition of ATP?

The in vivo experiment of MORC4 ATPaseCW triple basic patch mutant Supple. Fig 13 showed reduction of cells in S face, but it is no significant. Can you comment on that?

Reviewer 3, Comment 1: The nucleosome substrate presented in Figure 5C corresponds to the LexA, not to the Gal4 (it should be at least 30 bases between Cy3 and the Gal binding site). Also, on the Gal4 substrate, the distance between the Cy3-Cy5 is almost 90 Å (based on crude modeling, and assuming a B DNA conformation of the 30bp linker). Keeping this distance in mind, can a productive FRET signal be observed? See the attached figure (75 bp linker is not present in the model).

Author's response: Fig. 6c has been revised to show the Gal4-NCP model (it was LexA-NCP147).

Reviewer's response: Answered. The substrate makes more sense now.

Reviewer 3, Comment 2: Since MORC4 presents a high affinity for free double-strand DNA, why the nucleosome substrates have long DNA overhangs? The 75bp 3' linker is used in the cited paper to attach nucleosomes to the sensor surface. However, the assays are performed in solution. I recommend repeating the experiments with nucleosomes that contain no/minimal DNA overhangs.

Author's response: as suggested, we have examined binding of the ATPase and CW domains and the ATPaseCW cassette to NCP147. Binding of ATPaseCW was decreased 2-fold, whereas binding of the ATPase domain was decreased 5-fold, compared to binding of these constructs to NCP207. These results suggest that extra-nucleosomal DNA enhances binding of MORC4. The new data are shown in Suppl. Fig. 7 and the text on page 10 has been added/revised.

Reviewer's response: Answered. Probably the decrease in higher since the ATPase NCP147 since the curve is not saturated. To me S1/2 values on ATPaseCW are almost the same, but the ATPase has a difference of almost 10 times.

Reviewer 3, Comment 3: What is the minimum dsDNA length that can activate the ATPase activity of MORC4 ATPaseCW? Also, what is the minimum dsDNA length that can support MORC4ATPase DNA binding? The fully extended 601 DNA is significantly longer than the distance between the ATPase and CW domains. Therefore, multiple MORC4 can bind to this extensive DNA sequence. Can MORC4 ATPaseCW bind to the overhangs used in on the presented experiment?

Author's response: we have carried out EMSA experiments to determine the minimum dsDNA length-

it's ~30 bp. The data are shown in new Suppl. Fig. 6 and described on page 10.

Reviewer's response: I guess, the answer is yes. Since the overhangs are more than 30 bp.

Reviewer 3, Comment 4: Does Morc4 binding to nucleosome activate the ATPase activity? Also, since the affinity H3 peptide increase with K4 methylation. What is the affinity of MORC4 for H3K4me(1-3) nucleosomes?

Author's response: we have performed ATPase assays with the nucleosome (new Suppl. Fig. 1), the data demonstrate that the nucleosome stimulates the ATPase activity. We were unable to perform binding experiments with mono/di/tri-methylated NCPs because these nucleosomes are prohibitively expensive to us.

Reviewer's response: The point of this experiments was to measure if MORC4 ATPase activity is stimulated either by methylated nucleosomes or just nucleosomes.

Reviewer 3, Comment 5: The curve for MORC3 presented in Figure 5F shows significant variability in the measurement of the dFRET signal… This data should be removed.

Author's response: as suggested, these data have been removed.

Reviewer's response: Correction accepted

Reviewer 3, Comment 6: The FRET experiment in Figure 5E was performed with 0.3 mM ATP. However, the assay presented in Figure 3F was performed without ATP. It seems that the ATPase activity of MORC4 is not critical for the stabilization function, but without the appropriate controls, it is difficult to establish the mechanistic effect ATPase activity versus just the DNA or chromatin binding. Please include control experiments (With and without ATP).

Author's response: we have performed FRET assays with and without ATP (new data are shown in Fig. 6e) and found that the presence of ATP is essential (described on page 11).

Reviewer's response: Answered. That was an important control.

Reviewer 3, Comment 7: For the in-vivo experiments (Figure 6), the authors used a different set of mutants than in the biochemistry section. The authors should include the CWkrr mutant in the analysis. Also, there is no experimental evidence on the E56A ether disrupts ATPase activity or/and retains DNA binding. This data should be presented.

Author's response: we have tested the E56A mutant in ATPase activity assay and EMSA – it is catalytically inactive but retains the DNA binding activity (the new data are shown in Fig. 5f and Suppl. Fig. 10).

Reviewer's response: The data is convincing.

In Figure 6C, the authors observe a small increase in the percentage of cells in S phase when over expressed MORC4. The mutants used decreased the rate of cells in this phase, but the levels are not comparable to the mock control. So, what is the effect of the MORC4 double mutant on the cell cycle? Also, what is the impact of the MORC4 CWkrr on cell cycle? Do the authors know the distribution of MORC4 during the cell cycle?

We have repeated the cell cycle experiments and obtained very reproducible results (new data are shown in Fig. 7c and Suppl. Figs. 12 and 13). As suggested, we have included the dual and triple mutants. Due to a lack of verified commercially available antibodies we were unable to determine the cell cycle distribution of endogenous MORC4.

Reviewer's response: Supple. Fig 12, W435A/E56A show a very large error, higher that the value difference form WT MORC4, making this result difficult to explain. I was expecting similar percentage of S cell than the control. Redo it or removed.

Reviewer 3, Comment 8: In line 49, the authors quote that over expression of MORC4 in breast cancer tissue is linked with poor patient survival. Contrary, microRNAs targeting MORC4 decreased cell growth and activation of apoptosis (ref. 9). How the previous statement is compatible with what is presented in line 316, in which MORC4 plays a negative role in S-phase progression. The expected effect on tumor growth should be the opposite, particularly were cancer cells require more transcriptional activation for cell growth. The authors should clarify it with the proposed mechanism.

Author's response: MIR193-3p targets many other proteins known to regulate cell cycle including MYCN, KRAS, E2F6, DCAF7, HOXD13. Previous work has demonstrated a role for this miRNA as a tumor suppressor, and in breast cancer it is known to regulate KRAS expression (Grossi, Int J Genomics, 2017 PMID: 29038785). The previous work did not test MORC4 regulation of cell cycle or cell growth and using the miRNAs (and not just MORC4 expression) to do so has many compounding effects that would make interpreting those experiments difficult.

Reviewer 3, Comment 9:
Minor:
In Figure 1A, the authors should include the sequence registry and length of the constructs used in the study for clarity. – done

Reviewer's response: I still think that adding constructs length (biochem and x-ray) as a bar in the top of the diagram will be beneficial for the audience.

On cartoon representation of the MORC4 X-ray structure (Figure 1D), it is evident that there are several unstructured loops. Those regions should be represented in dashed lines. Also, residues mutated in this study should be shown in the context of the structure. – dashed lines are now included in Fig. 1d, and mutated residues are highlighted in Fig. 5a.

Reviewer's response: Well done.

Since the MORC family present significant identity (about 55 % between MORC4 and MORC3), the authors should show sequence alignment of the family, highlighting the sequence differences. – the alignment is shown in Suppl. Fig. 2.

Reviewer's response: Answered. It is much easier to compare differences between MORC4 and MORC3.

The authors should comment on the unsuccessful attempt to co-crystalize the MORC4 with H3K4me3 peptide, despite the promising peptide binding experiments. Also, it is not clear what is the effect of PTM peptides (particularly H3K4me) on MORC4 activity. – one possibility we were contemplating is that DNA needs to be present (although we did not obtain diffracting crystals in the presence of both DNA and the H3K4me3 peptide). To address this issue, we are collaborating with the Cramer lab on determining the structure of the ATPaseCW cassette in complex with NCP by cryo-EM.

Reviewer's response: Answered. It will be nice to add this to the discussion.

Regarding MORC4 binding to histones, it seems that just the methylation on H3K4 is the only significant event rather than the methylation state (me1, me2, or me3) (Figures 2A and 2C). Can be the potential peptide-binding site on the CW identified? There is a very distinct binding site configuration for me1, me2, or me3. – we have previously determined the crystal structures of the isolated CW domain of MORC3 in complex with the H3K4me3, H3K4me1 and H3unmod peptides (ref. 13). These peptides were bound in the same binding site, and K4 was positioned between two tryptophan residues in all complexes (please see figure above). The histone-binding site residues of MORC3 and MORC4 are highly conserved.

Reviewer's response: Answered. But my question was about, it will make more sense to mutate the tryptophan residues to alanine in order to reduce chromatin biding rather than unfold the hole CW with the W435A mutation. Also, this mutation is not included in figure 5a.

Which program or algorithm has been used to generate these panels on Figures 3F and 3G? APBS is recommended for this task. Since the CW structure misses some loop regions, have completed those for the figure purpose? – the electrostatic surface potential images were generated using APBS in Pymol with a range of –100/100 kT/e. The missing loops were completed. We have added this info in Fig. 3f, g legend.

Reviewer's response: I glad that you added the scale and clarified the software used , but I think the units that are reported are wrong. That is a too much range just a protein calculation. Indeed, I was able to repeat the Fig 3g. and the units are -5/5 kT/e (see the figure), that is similar on what you reported. That is reason the scale bar is important.

The authors should calculate the apparent Kds for the binding isotherms (gel shifts) presented in Figures 4E, 4F, 4G, and 4H. It will help to compare with other biochemistry experiments. Also, there is no description of what it is the meaning of the colored rectangle? 50% of the change in the signal of 601 DNA)? …Can apparent Kd been calculated for the experiment presented Supplementary Figure 4, to be compared with the DNA biding Kd presented Figure 5? – we did attempt to calculate apparent Kds but to avoid adding imprecise values decided not to use them.

Reviewer's response: That is OK. But since Kd values are not calculated, can you include the well in which you observe the half concentration of the free species (DNA or NCP).

The authors should label the rows of the image (DPI, mCheery?) in Figure 6. – done

Reviewer's response: OK.

In supplementary Figure 3, the complex seems to disappear at higher MORC4 ATPaseCWkrr. Does the protein/DNA complex remain on the wells or there is degradation? – it remains in the well.

Reviewer's response: Ok.

We thank the Editor and Reviewers for the insightful and very constructive comments, which were helpful in revising and strengthening this manuscript.

Reviewer 2, Comment 1: In particular, the fact that the MORC3 and MORC4 H3K4me binding site can be assumed to be similar based on sequence conservation helps to directly pinpoint the overlap of histone and ATPase binding sites in CW, which could be added to line 166. – as suggested, the phrase "…and that, similarly to MORC3[13-15], the ATPase-binding site and the histone-binding site of CW overlap" has been added to line 166.

I think the crux of this to understanding the mode of activation is to find out why the H3 tail is capable of binding the ATPaseCW cassette in MORC3 but not in MORC4 and how that is related to the DNA binding of the CW in MORC4? – this is incorrect, the ATPaseCW cassette of MORC4 binds to the H3 and H3K4me3 tails- please see Fig. 4a, c. What is different, that the DNA binding and catalytic activity of MORC4 are independent of histone binding, whereas the DNA binding and catalytic activity of MORC3 depend on it. We discuss these differences in detail on pages 5, 6, 13 and 14.

…in line 325/326 it is mentioned to be essential for activity of MORC4 without any proof, where in the case of MORC3 more data is available – this sentence has been revised to: "While both DNA binding and dimerization are essential for the ATPase activity of MORC3, mutation…"

Reviewer 2, Comment 2: The new data on the DNA binding site within the ATPase domain are unclear. It is concluded that fig. 5b and 5c show most reduction in DNA binding, while, without any quantitation of the free DNA band intensity available, the mutants in Fig 5d and 5e seem to have the strongest reduction. – yes, a weak decrease in intensity of the free DNA band indicates that the mutants in Fig. 5b, c have a reduced DNA binding activity compared to the WT protein. The strongest reduction of the free DNA band intensity indicates that the mutants in Figs. 5d and 5e bind DNA well.

Reviewer 2, Comment 3: The FRET experiments lead to two conflicting conclusions: line 257/258 "This suggests that binding of the ATPaseCW cassette itself does not shift the equilibrium." but in line 265/266: "Collectively, these data reveal that binding of the MORC4 ATPaseCW cassette to the nucleosome shifts the unwrapping-wrapping equilibrium toward the wrapped state". This needs to be explained. – the first two sentences of this paragraph have been revised, thank you for catching it.

Other issues:
- line 104-111: a supplemental figure would be highly helpful here – Suppl. Fig. 2 has been added
- line 174: "band corresponding to the complex of CW with 601 DNA" is better rephrased to " a band smear corresponding to complexes of CW with 601 DNA" as multiple CW will associate to the long DNA substrate explaining the smeared appearance. – rephrased as suggested
- line 176: "MORC3 was incapable of binding to 601 DNA" is better toned down since there is clear reduction of the free DNA bind indicating binding, even though perhaps the complexes precipitated or got stuck in the well which is not clearly visible in the picture – revised to "was largely incapable"
- line 203: "(both ATPase and CW domains are involved in this interaction)" a reference is needed here – these are our current results

Reviewer 3, Comment 1: there are some minor comments that still need to be addressed

In page 4-5, when the structure is described, but there is no figure for that. I recommend to add secondary structure to the top of alignment in Fig. Supple.2, including the secondary structure names (ej. α1, β8, etc). – as suggested, the secondary structure elements have been added to Suppl. Fig. 3.

In the section ‘Extensive DNA binding site of MORC4 ATPaseCW Cassette”, the authors identify two positive clusters in the structure surface, and speculate that since there are 40 Å away the surface is large. Authors should calculate the area and compare it with other DNA binding proteins. – thank you for this suggestion, though by doing so we would overstate the results. We don’t know size or the exact position of the DNA-binding site; we hope a cryo-EM structure will provide this information.

The experiment of MORC4 ATPaseCW binding to Nucleosomes (Data not shown), Does the experiment was performed with the addition of ATP? – without ATP, and we removed it.

The in vivo experiment of MORC4 ATPaseCW triple basic patch mutant Supple. Fig 13 showed reduction of cells in S face, but it is no significant. Can you comment on that? – we have added the comment on page 12 “… K460A/R462A/R463A mutant, which suggested that the disruption of the DNA binding activity of CW is not enough (Supplementary Fig. 13).”

Supple. Fig 12, W435A/E56A show a very large error, higher that the value difference form WT MORC4, making this result difficult to explain. I was expecting similar percentage of S cell than the control. Redo it or removed. – we have removed this figure.

I still think that adding constructs length (biochem and x-ray) as a bar in the top of the diagram will be beneficial for the audience. – we believe that adding bars in Fig 1A would make the overall figure too busy and destruct from the major point of this figure- the catalytic activity and the structure.

the W435A mutation. Also, this mutation is not included in figure 5a. – W435 is now shown in Fig 5a.

I glad that you added the scale and clarified the software used , but I think the units that are reported are wrong. – the units have been corrected.

But since Kd values are not calculated, can you include the well in which you observe the half concentration of the free species (DNA or NCP). – we attempted to do so, however not a single gel has a well with 50% reduction of the free species- for example, the closest to 50% numbers are between 45% and 57% for the gels shown in Fig 4e-h.
